# Serum ferritin in children with Attention Deficit Hyperactivity Disorder (ADHD): A systematic review and meta-analysis

**DOI:** 10.1371/journal.pone.0359790

**Published:** 2026-10-05

**Authors:** Solomon Gedfie, Muluken Gashaw, Abebe Adisu, Alembante Bazezew, Abdu Jemal, Mengistu Tesfa Alula, Bewuketu Belete Alemu, Marye Nigatie, Zewdu Tefera, Bayih Ejigu Yimer, Yalewayker Gashaw, Agenagnew Ashagre, Wagaw Abebe, Woldeteklehaymanot Kassahun

**Affiliations:** 1 Department of Medical Laboratory Sciences, College of Health Sciences, Woldia University, Woldia, Ethiopia; 2 Department of Sport Science, College of natural Science, Woldia University, Woldia, Ethiopia; Taipei Veterans General Hospital, TAIWAN

## Abstract

**Background:**

Attention-deficit/hyperactivity disorder (ADHD) is a common childhood neurodevelopmental disorder of uncertain etiology. Iron is crucial for brain function and dopamine regulation, but prior studies examining its association with ADHD have produced inconsistent results. This systematic review and meta-analysis aimed to compare serum ferritin concentrations in children with ADHD versus healthy controls.

**Method:**

This systematic review and meta-analysis was conducted in accordance with the Preferred Reporting Items for Systematic Reviews and Meta-Analyses (PRISMA) guidelines. A comprehensive literature search was conducted across PubMed/MEDLINE, Scopus, Science Direct, and Google Scholar to identify eligible studies. Statistical analyses were performed using STATA version 17, and a random-effects model was applied to estimate the pooled mean difference (MD) and standardized mean difference (SMD). Heterogeneity across included studies was assessed using Higgins’ I^2^ test. Publication bias was evaluated visually using funnel plots and statistically via Egger’s weighted regression test.

**Results:**

A total of 7643 articles were identified, of which 21 studies (including 4,058 ADHD cases and 4,533 controls) were included in the final meta-analysis. The pooled mean difference (MD) in serum ferritin levels between children with ADHD and healthy controls was −9.74 (95% CI: −16.50, −2.98). Subgroup analyses were conducted to explore heterogeneity. By continent, the highest pooled MD was observed in Africa (−19.28, 95% CI: −40.73, 2.17) and the lowest in North America (−1.42, 95% CI: −6.98, 4.13). By publication year, studies published before 2015 showed a pooled MD of −1.94 (95% CI: −2.31, −1.58), compared to −19.43 (95% CI: −34.37, −4.49) for those published after 2016. By sample size, smaller studies (<199 participants) yielded a higher MD (−12.12, 95% CI: −21.91, −2.34) than larger studies (≥200 participants; −7.64, 95% CI: −17.33, 2.05). By study design, the pooled MD was −1.95 (95% CI: −2.31, −1.59) for case-control studies and −14.85 (95% CI: −27.07, −2.63) for comparative cross-sectional studies. In the subgroup analysis based on ADHD diagnostic criteria, the DSM-IV-TR criteria yielded the largest mean difference (−16.56, 95% CI: −28.23, −4.89).

**Conclusion:**

Lower serum ferritin levels (pooled MD: −9.74) are associated with ADHD in children. Accordingly, screening for serum ferritin and enhanced iron supplementation should be considered in this population. Future longitudinal studies are needed to clarify the causal relationship between iron deficiency and ADHD severity.

## Introduction

Attention deficit hyperactivity disorder (ADHD) is a neurodevelopmental brain disorder marked by a persistent and maladaptive pattern of inattention, as well as hyperactivity or impulsivity, that impairs functioning or development [1,2]. Among school-aged children, ADHD has a prevalence of 5–10%, and in 30–50% of these cases, the disorder continues into adolescence and adulthood [3]. Worldwide, the estimated prevalence of ADHD in children below 18 years old varies between 8% and 12%, with boys showing higher rates than girls [4,5]. In children and adolescents, ADHD creates challenges in academic settings, studying, and social interactions. These difficulties result in developmental, educational, and social disadvantages, as well as a significant financial strain on both healthcare and education systems. Throughout life, ADHD serves as an ongoing risk factor for various comorbidities, including psychiatric conditions like depression, anxiety, and behavioral disorders, along with accidents, impaired social functioning, and obesity [2,6].

While the exact cause of ADHD remains unclear, the prevailing understanding is that it arises from a complicated network of interactions involving genetic, environmental, and social factors [7]. Thus, the suggested causes including prenatal and perinatal risks, genetic factors, and neurobiological impairments may each contribute to the underlying pathophysiology of ADHD, though their roles can vary from one individual to another [3]. Nevertheless, a wide range of studies employing diverse research methods have pointed to dopamine as a crucial factor in the pathophysiology of ADHD. Researchers have examined the link between ADHD and genes that regulate neurotransmitters such as dopamine, norepinephrine, serotonin, and gamma-aminobutyric acid (GABA). Among these, dopamine is likely to play a central role, given its involvement in controlling psychomotor activity and executive functions two core clinical features seen in individuals with ADHD [8].

Numerous molecular genetic studies on ADHD have focused on genes linked to dopamine function, with particular attention given to the dopamine D4 receptor gene and the dopamine transporter gene (DAT1). The connection between the dopamine transporter and ADHD is especially significant, as this site serves as the primary target for commonly prescribed ADHD medications, including methylphenidate, pemoline, and dexamphetamine. Additionally, iron acts as a cofactor for tyrosine hydroxylase, the enzyme that limits the rate of dopamine synthesis [9]. As a result, iron levels stored in the brain could impact dopamine production, which in turn may influence a range of behavioral characteristics especially those observed in individuals with ADHD [10,11].

Studies into the neurobiological underpinnings and treatment of ADHD indicate that dietary factors including glucose metabolism, fatty acid metabolism, and deficiencies in vitamins or minerals can influence brain function and play a role in the onset of the disorder [9,12,13]. Among these nutritional factors, iron deficiency (ID) has received particular attention because of iron’s essential role in regulating dopaminergic activity, which is linked to both the pathogenesis and symptoms of ADHD [14].

In a study by Sever et al., children with ADHD who were given iron supplements showed significantly increased serum ferritin levels and reduced scores on ADHD symptom scales, suggesting that even non-anemic individuals may benefit from iron therapy. This outcome spurred additional investigations into the relationship between ID and ADHD. However, findings from subsequent studies have often been inconsistent. For example, while some researchers reported lower average serum ferritin concentrations in children with ADHD compared to their healthy counterparts, other studies were unable to replicate these results [14–16].

One study found that the severity of ADHD is inversely related to serum ferritin levels [15], In contrast, another study [14] used magnetic resonance imaging (MRI) to compare brain iron levels between children with ADHD and healthy controls, finding that estimated brain iron was significantly lower in the bilateral thalami of those with ADHD. Likewise, one study [16] reported that medication-naïve individuals with ADHD exhibited lower estimated brain iron levels in both the striatum and thalamus compared to healthy controls. Nevertheless, other researchers have not identified any link between serum ferritin levels and ADHD [17,18].

Iron plays an essential role in numerous biological processes, especially during early development. Despite its importance in brain growth and function, the precise mechanisms through which iron operates and the full scope of its involvement in normal brain physiology remain unclear. According to the World Health Organization, ID is the most widespread nutritional deficiency globally [6].

The synthesis of dopamine is regulated by the enzyme tyrosine hydroxylase, which converts tyrosine into L-dopa; L-dopa is then transformed into dopamine through a decarboxylation process. Iron stored in the brain influences this dopamine production, thereby impacting various behaviors particularly in children with ADHD. One study found that brain ferritin levels were significantly lower in children with ADHD compared to control subjects [14].

In a study by Juneja et al., low serum ferritin levels were found in 92% of children with ADHD. Similarly, Konofal’s research reported low ferritin levels in 84% of children with ADHD, compared to only 18% of children without the disorder. Furthermore, a randomized controlled trial (RCT) that compared iron supplementation with a placebo over 12 weeks found a significant decrease in ADHD rating scores among children receiving iron [19].

In recent years, multiple studies have been conducted to assess whether serum ferritin levels serve as a reliable indicator of iron stores in body tissues including the brain in non-anemic children with ADHD. However, only a small number of these studies have identified a link between low ferritin levels and the presence of ADHD [15,17,20].

Although numerous studies have examined serum ferritin levels in children with ADHD versus healthy controls, results remain inconsistent. Some report significantly lower ferritin levels in children with ADHD, while others find no difference. Given these conflicting findings, determining the overall association between serum ferritin and ADHD is essential. Thus, we conducted a systematic review and meta-analysis to estimate this association.

## Method

### The study protocol and registration

This systematic review and meta-analysis were conducted based on the Preferred Reporting Items for Systematic Review and Meta-Analysis (PRISMA) guideline [21]. The protocol for this systematic review and meta-analysis has been registered in the PROSPERO, an International Prospective Register of Systematic Reviews with a registration number of: **CRD420261420475**. **Review question**: children (**population**) with ADHD (**exposure**) had lower levels of mean serum ferritin (**outcome**) than healthy controls (**comparison**)?

### Search strategy

Data were retrieved by searching for published articles in databases, such as Scopus, PubMed, Science direct, Google Scholar search engine, and other institutional repositories. There was no time restriction during article searching, articles published up to June 10, 2026, were included. The search terms were used separately and in combination using the Boolean operators like “OR” or/and “AND”. The search terms were “Iron deficiency”, “serum ferritin”, “serum iron”, “Iron deficiency anemia”, “Iron parameters”, “ADHD”, “Attention deficit hyperactivity disorder”, “Children”. The search strings used in PubMed was:- (((((((((((((serum ferritin) OR (ferritin level)) OR (serum iron)) OR (iron deficiency)) OR (ID)) OR (iron deficiency anemia)) OR (IDA)) AND (children)) AND (Attention Deficit Hyperactivity Disorder)) OR (ADHD)) OR (Attention Deficit Disorder)) OR (ADD)) OR (Hyperkinetic Disorder)) OR (Hyperkinesis). We identified additional articles by conducting a manual search and by examining the references cited in pertinent papers. The search strategy and number of articles retrieved from the searched databases are depicted in the additional file (**S1 Table in** **S1 File**).

### Inclusion and exclusion criteria

This review included peer-reviewed articles as well as studies deposited in institutional electronic repositories or registries. Eligible studies involved children with ADHD and healthy controls, utilized case-control, comparative cross-sectional, or cohort designs, and reported serum ferritin levels. Moreover, studies were included only if they satisfied two further conditions: serum ferritin assessment in children with ADHD relative to non-ADHD controls, and ADHD diagnosis established according to standardized protocols. Only articles published in English before June 10, 2026, were included. The review excludes case reports, case series, studies involving children with psychiatric disorders other than ADHD, known hematological abnormalities, or iron homeostasis disorders. Conference abstracts, reviews, and animal studies were also excluded.

**Study selection and quality assessment**: To organize search outcomes and to remove duplicate articles the searched articles were imported into EndNote X 21 (Thomson Reuters, New York, USA). Two investigators independently selected the studies. Initially, clearly irrelevant studies were excluded by scanning titles and abstracts. The full texts of the remaining articles were then evaluated carefully according to our eligibility criteria. Where required, any disagreement on eligibility for inclusion was resolved by a third author. Study quality was rated by two authors using the Joanna Briggs Institute (JBI) critical appraisal tools, which is recommended for quality assessment of cohort and case-control studies, and has a maximum score of ten. Studies scoring 7–10, 4–6, and 0–3 are regarded as high quality, moderate quality, and low quality, respectively [22]. The quality appraisal guideline contains ten evaluation domains or categories to evaluate the internal and external validity. The items are: (a) were the groups comparable other than the

Presence of disease in cases or the absence of disease in controls?, (b) sampling frame Were cases and controls matched appropriately?, (c) Were the same criteria used for identification of cases and controls?, (d) Was exposure measured in a standard, valid and reliable way?, (e) Was exposure measured in the same way for cases and controls, (f) Were confounding factors identified?, (g) Were strategies to deal with confounding factors stated?, (h) Were outcomes assessed in a standard, valid and reliable way for cases and controls?, (i) Was the exposure period of interest long enough to be meaningful?, and (j) Was appropriate statistical analysis used?. Each category was evaluated as Yes, No, unclear and not applicable. Unclear was considered as a high risk of bias.

**Data extraction**: Two reviewers independently extracted key data, including first author, publication year, country, study design, age (mean ± SD), mean and standard deviation of serum ferritin for cases and controls, and sample sizes for both groups. Extracted information was compiled into an MS Excel spreadsheet. Any disagreements were resolved by consensus or through discussion involving a third author (**Table 1**).

**Table 1:** Characteristics of included studies for Serum Ferritin in Children with Attention Deficit Hyperactivity Disorder (ADHD).

Author	Publication year	country	Study design	Total sample size (ADHD/CONTROL)	Mean age	Mean Serum ferritin ADHD (ng/ml)	Mean Serum ferritin controls (ng/ml)	ADHD Diagnostic method	Quality score
Eli Lahat et al. [31]	2011	Israel	CCS	67/46	8.8 ± 2.7	20.8 ± 12.3	31.2 ± 13.2	DSM-IV-TR	8
Bahgat KAE et al.[27]	2022	Egypt	CCS	40/20	8.15 ± 1.35	15.86 ± 5.55	89.4 ± 26	DSM-IV-TR	8
Wirantari NP et al. [36]	2020	Indonesia	CC	25/25	5.4 ± 1.8	43.1 ± 15.4	122.2 ± 47.7	DSM-IV-TR	7
Bener A [33].	2015	Turkey	CC	630/630	11.54 ± 3.83	36.26 ± 5.39	38.14 ± 5.61	DSM-IV-TR	7
Donfrancesco R et al.[18]	2013	Italy	CC	101/93	7.23 ± 3.66	33.01 ± 17.79	33.14 ± 18.73	K-SADS-PL	8
Menegassi M et al.[38]	2010	Brazil	CC	62/62	8.9 ± 2.5	54.2 ± 17.2	59.3 ± 21	K-SADS-E	7
El-Saadany NZH et al.[28]	2022	Egypt	CC	42/42	7.63 ± 2.637	60.6 ± 5.94	96.94 ± 13.29	DSM-IV-TR	7
Öztürk Y et al.[34]	2020	Turkey	CCS	99/106	8.19 ± 3.67	32.83 ± 17.11	44.66 ± 38.77	DSM-IV-TR	7
Konofal Eet al [17].	2004	France	CCS	53/27	9.2 ± 2.2	23 ± 13	44 ± 22	DSM-IV-TR	7
Luzuko Magula et al. [35]	2019	South Africa	CCS	156/89	9.5 ± 3.2	42.96 ± 25.28	49.28 ± 40.56	DSM-IV-TR	8
Millichap JG et al.[20]	2006	USA	CCS	68/371	8.5 ± 3.1	34.8 ± 17.73	36.3 ± 18.2	Clinical interview	8
Ali MWE et al.[29]	2025	Egypt	CC	100/100	8.7 ± 3.6	37.4 ± 6.8	38.4 ± 6.4	DSM-IV-TR	8
Oner O et al.[19]	2008	USA	CCS	52/52	9.9 ± 2.1	30.6 ± 15.4	33.01 ± 17.79	K-SADS-PL	7
Tang CY et al.[32]	2022	China	CCS	1565/1997	7.92 ± 1.85	36.63 ± 20.32	71.66 ± 51.99	DSM-IV-TR	9
Berner A et al.[37]	2014	Qatar	CC	630/630	8.92 ± 5.85	36.26 ± 5.39	38.14 ± 5.61	K-SADS-E	10
Juneja M et al.[15]	2010	India	CC	25/25	8.44 ± 1.68	6.04 ± 3.85	48.96 ± 41.64	DSM-IV-TR	7
Kwon HJ et al.[40]	2011	Korea	CC	48/48	6.98 ± 0.397	35.8 ± 16.6	37.1 ± 18.3	Clinical interview	7
Mahmoud MM et al.[30]	2011	Egypt	CC	58/15	8.6 ± 1.8	24.8 ± 14.1	32.6 ± 18.7	K-SADS-E	7
Cortese S et al.[14]	2011	France	CC	15/28	9.9 ± 1.5	32.4 ± 13.4	51.6 ± 16.4	K-SADS-PL	7
Adisetiyo V et al.[16]	2014	USA	CC	22/27	12.6 ± 2.8	50.8 ± 25.2	38.2 ± 22.8	DSM-IV-TR	7
Percinel I et al.[39]	2016	Finland	CC	200/100	11.0 ± 2.4	27.9 ± 15.3	30.8 ± 17.5	Clinical interview	9

***CC=case-control, CCS=Comparative cross-sectional***

**Statistical analysis**: The data were checked for completeness in Microsoft Excel before being transferred to STATA version 17 for final analysis. Due to the high heterogeneity between studies, we chose a random effects model to calculate the pooled SMD [23]. The standardized mean difference (SMD) was used to assess the association between serum ferritin level and ADHD. Forest plots were generated to display the overall pooled SMD, along with the relative weight assigned to each included study. The degree of heterogeneity was assessed using Higgins’ I² statistic, with I^2^ values categorized as low (25%), moderate (50%), and high (75%) [24]. We also used Galbraith radial plots to explore which studies may be contributing to heterogeneity. To explore potential sources of heterogeneity, sub-group analyses were performed based on country, year of publication, study design, and sample size. A sensitivity analysis was conducted by sequentially excluding individual studies to assess whether any single study had a significant influence on the pooled estimate. To assess potential publication bias, funnel plots were visually inspected, and Egger’s weighted regression test was performed. We also performed random-effects meta-regressions. In Egger’s test, a p-value < 0.05 was considered indicative of statistically significant publication bias [25].

### Ethics approval and consent to participate

As this study is a systematic review and meta-analysis of previously published data, it did not involve direct patient contact or primary data collection. Therefore, ethical approval and informed consent were not required.

## Results

### Literature search and identified studies

This systematic review and meta-analysis included articles on serum ferritin level among children with ADHD and healthy controls. Pubmed/MEDLINE, ScienceDirect, Scopus, and Google Scholar were among the databases used in the searching strategy. The review identified 7643 studies. After removing 521 duplicate records, 7085 studies were excluded following title and abstract screening. After screening 37 full-text articles for eligibility, 16 were excluded as they did not report the relevant outcome. Consequently, 21 studies met all inclusion criteria and were included in the final meta-analysis (Fig 1).

**Fig 1:**
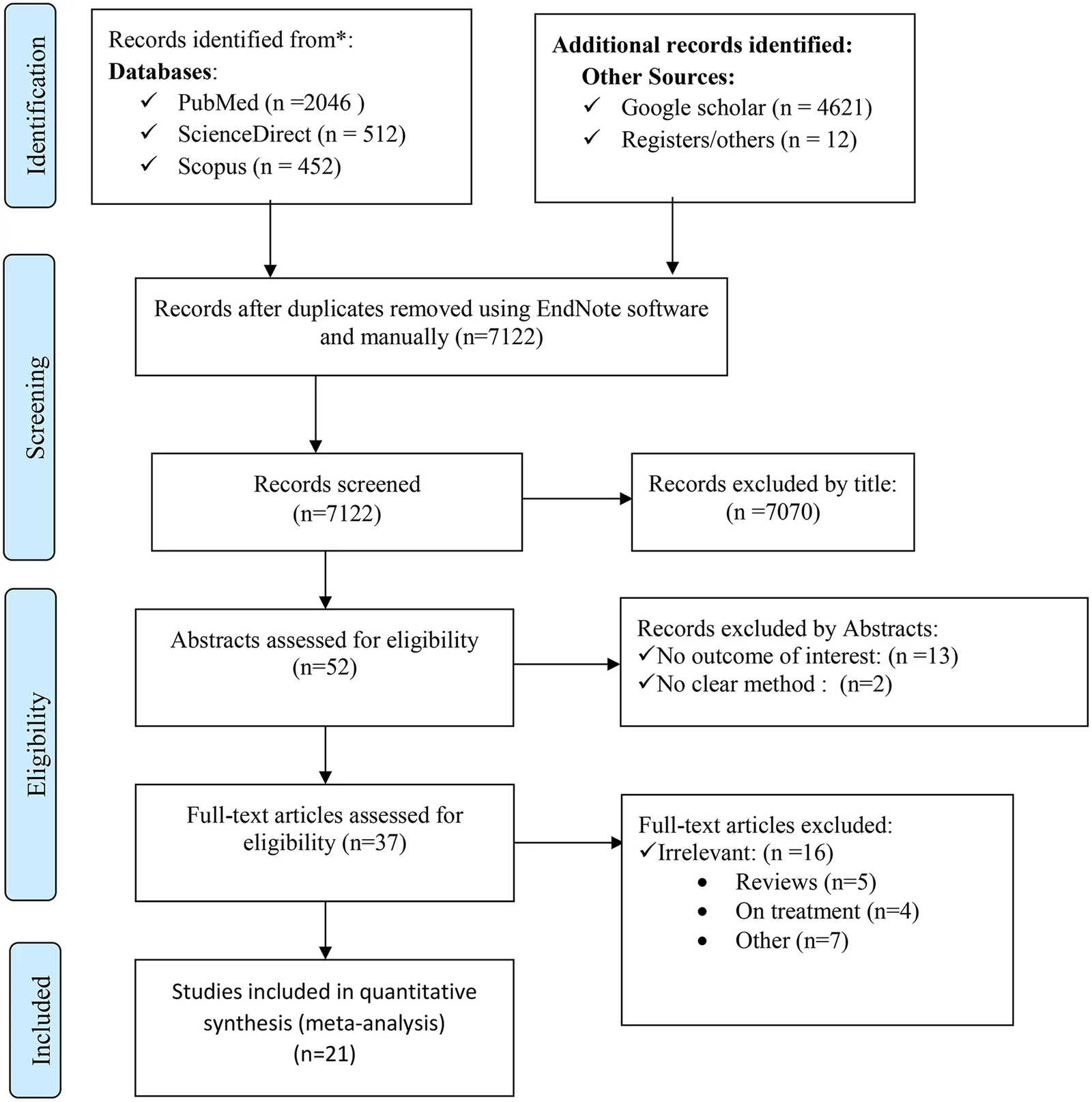
PRISMA flow diagram showing the results of the search and reasons for exclusion on systematic review and meta-analysis of Serum Ferritin in Children with Attention Deficit Hyperactivity Disorder (ADHD) [26].

### Characteristics of included studies

A total of 21 studies met the inclusion criteria for this systematic review and meta-analysis Among the included studies, four were conducted in Egypt [27–30], one were conducted in Israel [31], two were conducted in France [14,17], one conducted in China [32], two study were conducted in Turkey [33,34], one in South Africa [35], three were in USA [16,19,20], one in Indonesia [36], one in Qatar [37], one in Brazil [38], one in Italy [18], one in India [15], one in Finland [39] and one study in Korea [40] (**Table 1**).

### Methodological quality and risk-of-bias assessment

All potential papers were assessed for quality using the recommended quality assessment tool, the Joanna Briggs Institute (JBI), for prevalence data/observational studies. Two reviewers independently performed a critical appraisal of each paper. This involved a thorough examination using the JBI appraisal domains as follows: (a) were the groups comparable other than the Presence of disease in cases or the absence of disease in controls?, (b) sampling frame Were cases and controls matched appropriately?, (c) Were the same criteria used for identification of cases and controls?, (d) Was exposure measured in a standard, valid and reliable way?, (e) Was exposure measured in the same way for cases and controls, (f) Were confounding factors identified?, (g) Were strategies to deal with confounding factors stated?, (h) Were outcomes assessed in a standard, valid and reliable way for cases and controls?, (i) Was the exposure period of interest long enough to be meaningful?, and (j) Was appropriate statistical analysis used?. Each category was evaluated as Yes, No, unclear and not applicable. Unclear was considered as a high risk of bias. If consensus between the two independent reviewers could not be reached, a third reviewer was engaged to resolve any disagreements and facilitate consensus. Studies scoring 50% or higher on the final quality assessment were eligible for inclusion in this systematic review and meta-analysis.

### Serum Ferritin level among ADHD and Healthy controls

Serum ferritin levels were significantly lower in patients with ADHD compared with healthy controls, with a pooled MD estimate from 21 studies of −9.74 (95%CI = −16.50 to −2.98) indicating a negative association between serum ferritin and ADHD. However, there was significant statistical heterogeneity across studies (I^2^ = 99.16%, p = 0.00). (Fig 2). Additionally, the pooled standardized mean difference (SMD) across 21 studies comparing cases and controls was −10.4 (95% CI: −16.17, −4.64), as shown in (Fig 3).

**Fig 2:**
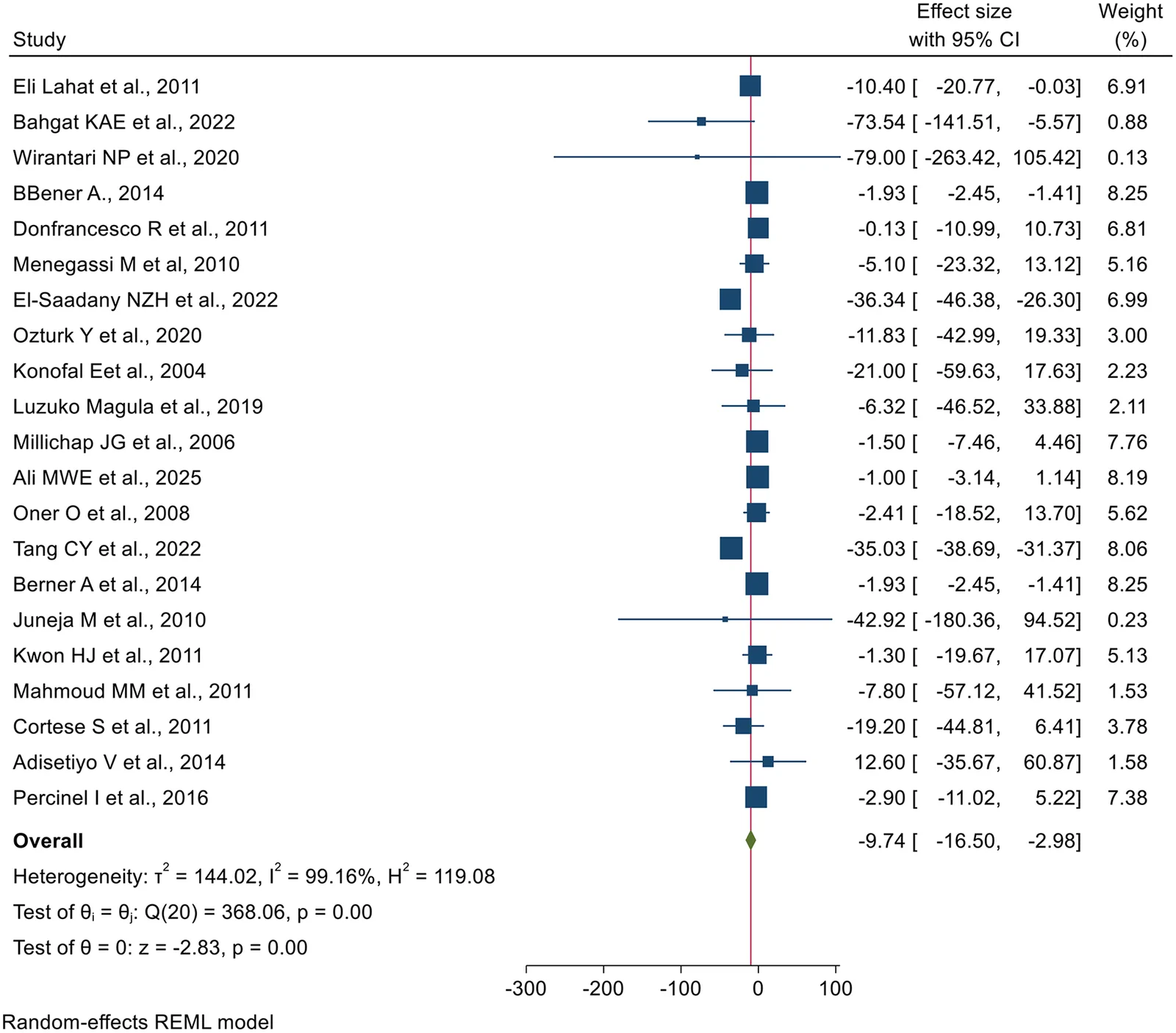
Forest plot showing the pooled MD of serum ferritin levels among children with ADHD and controls.

**Fig 3:**
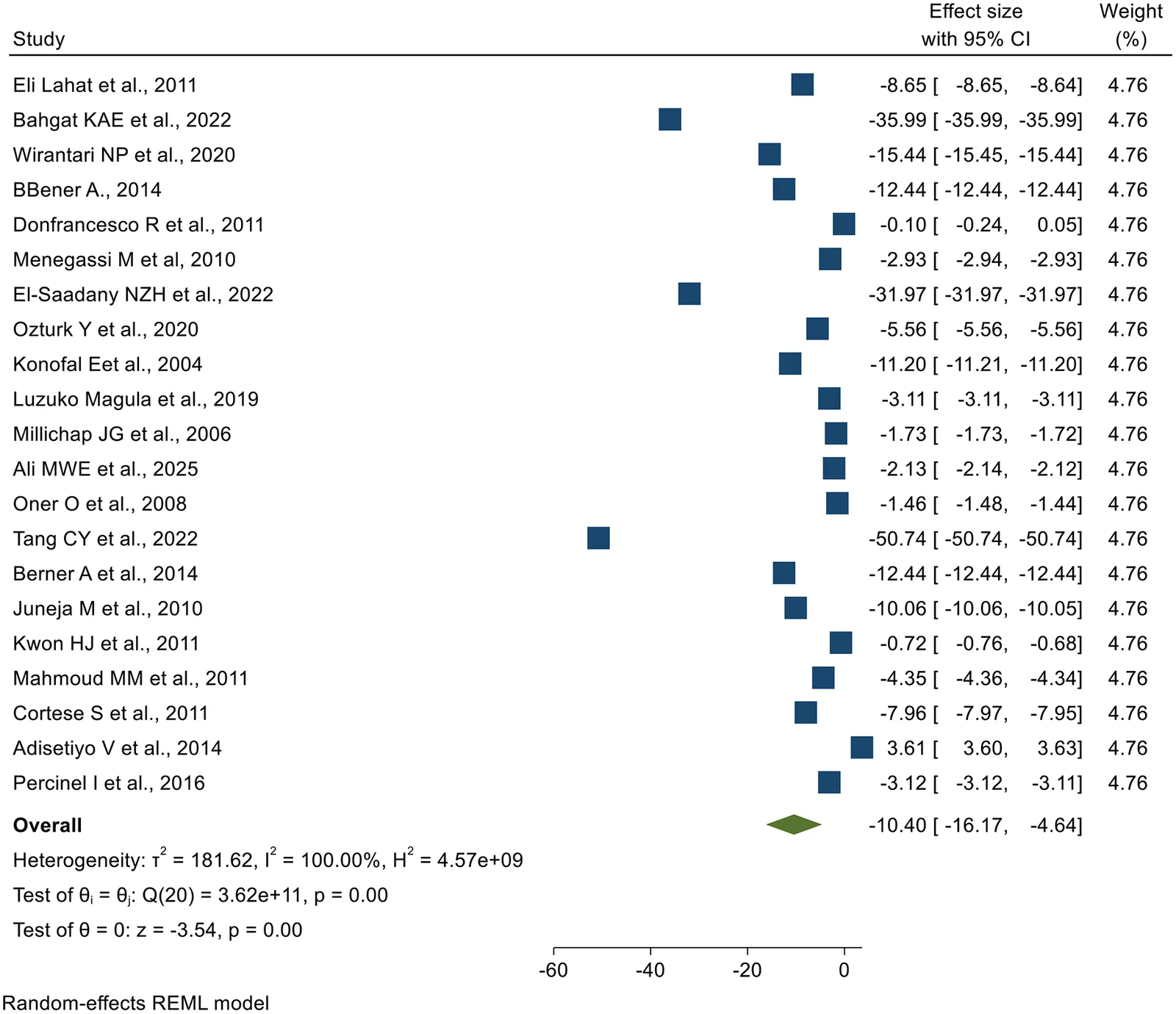
Forest plot showing the poorest plot showing the pooled SMD of serum ferritin levels among children with ADHD and controls.

Given the significant heterogeneity observed across the included studies (I^2^ = 99.16%%, p = 0.000), subgroup analyses were performed to explore potential sources of heterogeneity. The sub-group were conducted based on: country, publication year, study design, and sample size. The results showed significant variation in the pooled SMD across regions with Africa having the highest estimate at −19.28 (95% CI: −40.73, 2.17), followed by Asia at −15.03 (95% CI: −36.56, 6.50). In contrast, North America had the lowest SMD at −1.42 (95% CI: −6.98, 4.13) (Fig 4). When stratified by publication year, the pooled SMD of serum ferritin level was −1.94 (95% CI: −2.31, −1.58) for studies published before 2015, without significant heterogeneity (I^2^ = 0.00%, p 0.90). Among studies published in 2016 or later, the SMD was at −19.43 (95% CI: −34.37, −4.49), though substantial heterogeneity (I^2^ = 96.07%, p = 0.00) (Fig 5).

**Fig 4:**
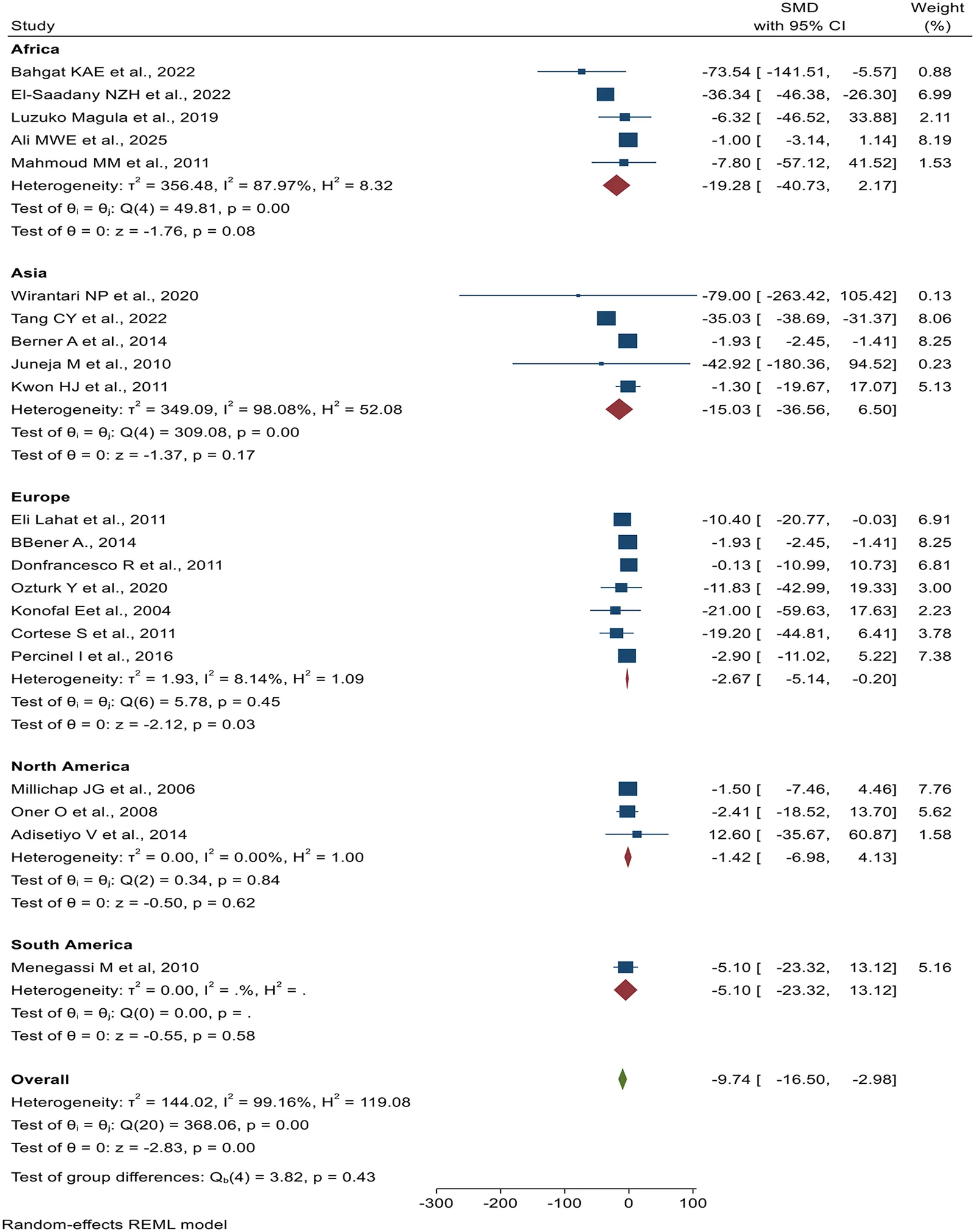
Forest plot showing sub-group analysis of the pooled MD of serum ferritin levels among children with ADHD and controls by Country.

**Fig 5:**
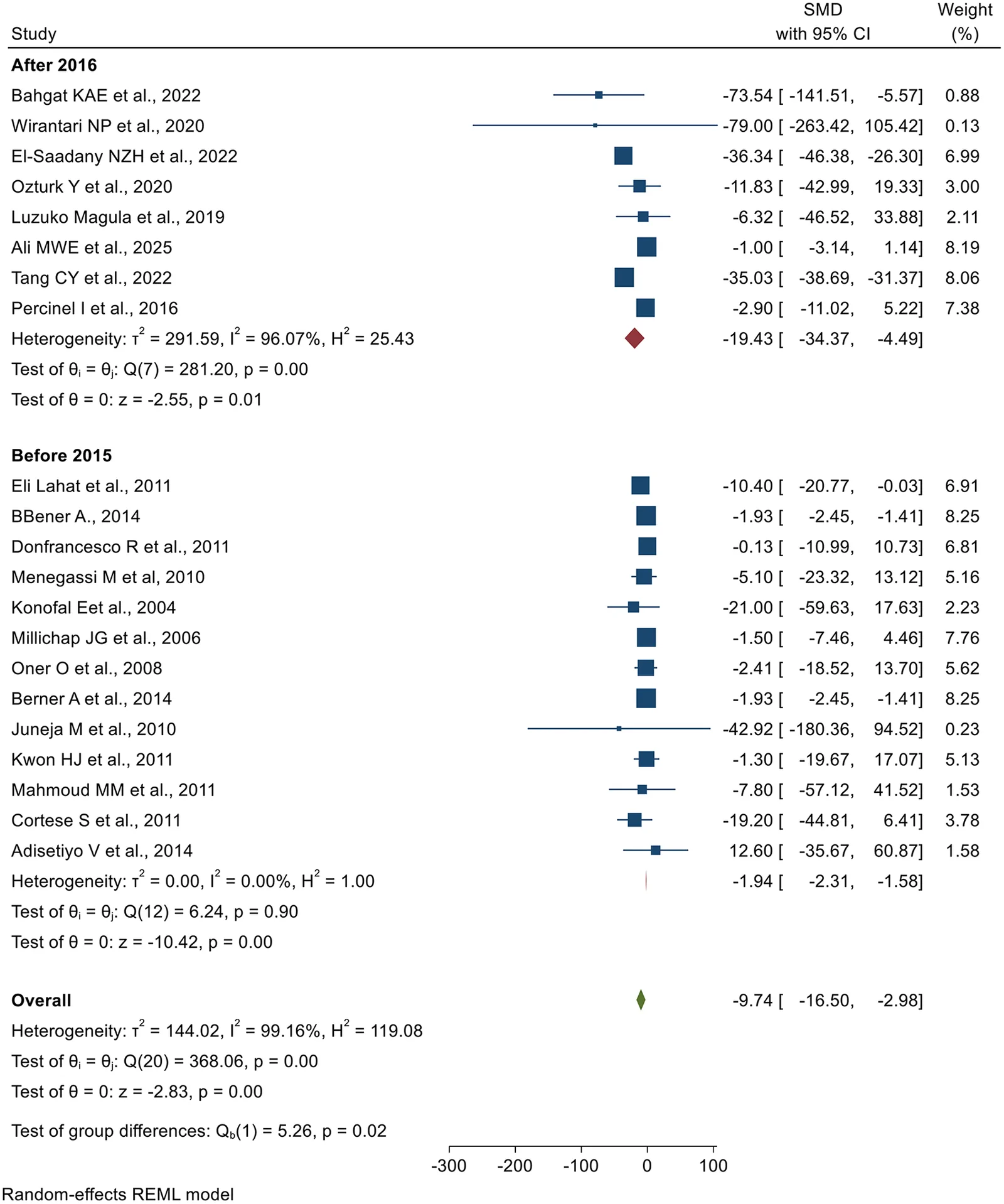
Forest plot showing sub-group analysis of the pooled MD of serum ferritin levels among children with ADHD and controls by year of publication.

Subgroup analysis by study design revealed a −1.95 (95% CI: −2.31, −1.59) pooled SMD among case control studies and −14.85 (95% CI: −27.07, −2.63) among cross-sectional studies, reflecting variability across studies (Fig 6). Another subgroup analysis by sample size demonstrated notable variations in pooled SMD. Studies with smaller sample sizes (<199 participants) yielded a higher pooled SMD of −12.12 (95% CI: −21.91, −2.34), though with considerable heterogeneity (I^2^ = 60.29%, p = 0.000). Conversely, studies with larger sample sizes (≥200 participants) showed a lower prevalence of −7.64 (95% CI: −17.33, 2.05), with heterogeneity (I^2^ = 99.73%, p = 0.000) (Fig 7). Subgroup analysis based on ADHD diagnostic criteria revealed substantial heterogeneity in the pooled mean difference (MD). The MD was −1.95 (95% CI: −6.60, 2.70) for clinical interviews, −16.56 (95% CI: −28.23, −4.89) for DSM-IV-TR, −1.96 (95% CI: −2.45, −1.41) for K-SADS-E, and −2.86 (95% CI: −11.36, 5.63) for K-SADS-PL. Notably, the DSM-IV-TR criteria yielded the largest mean difference (Fig 8).

**Fig 6:**
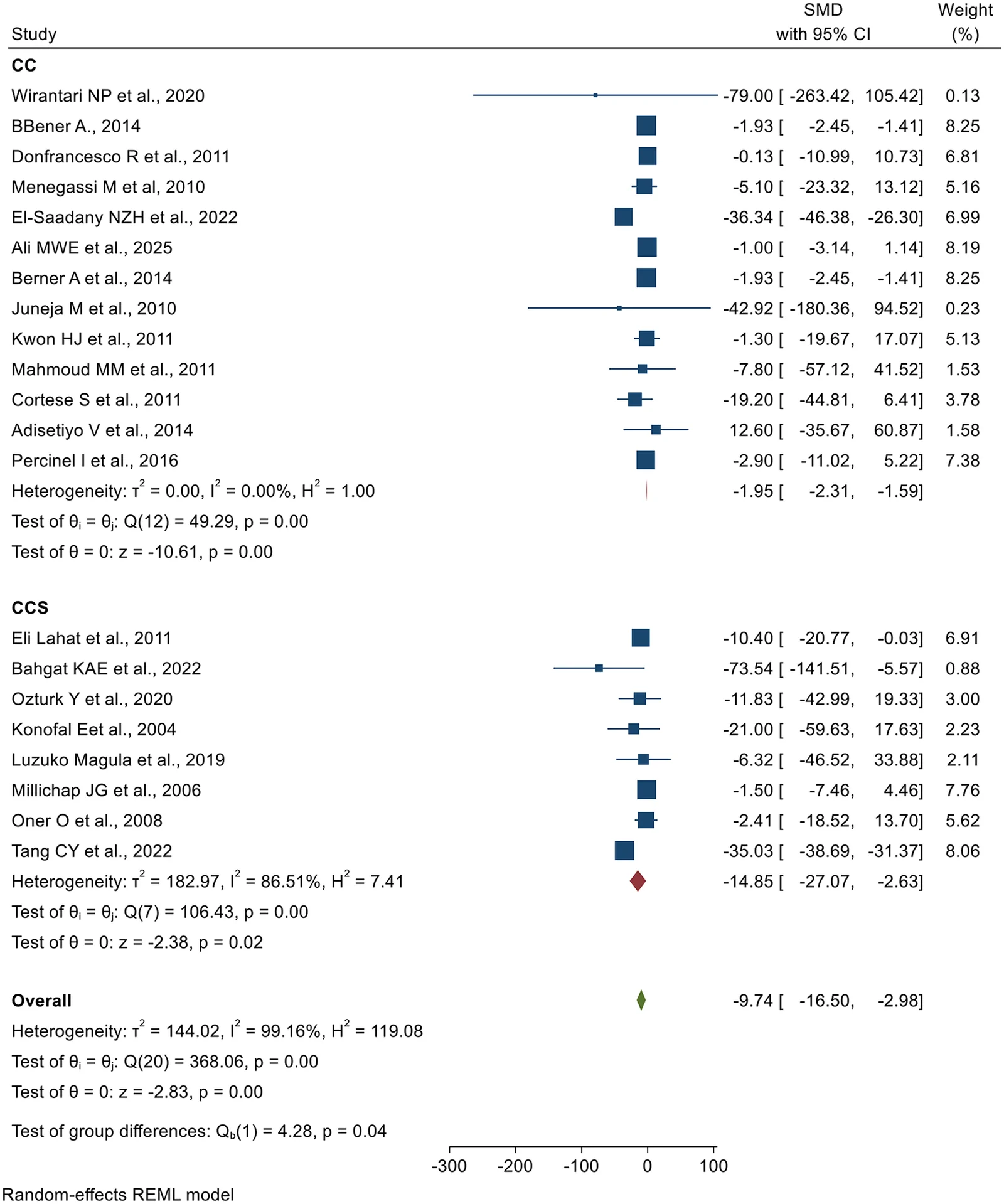
Forest plot showing sub-group analysis of the pooled MD of serum ferritin levels among children with ADHD and controls by study design.

**Fig 7:**
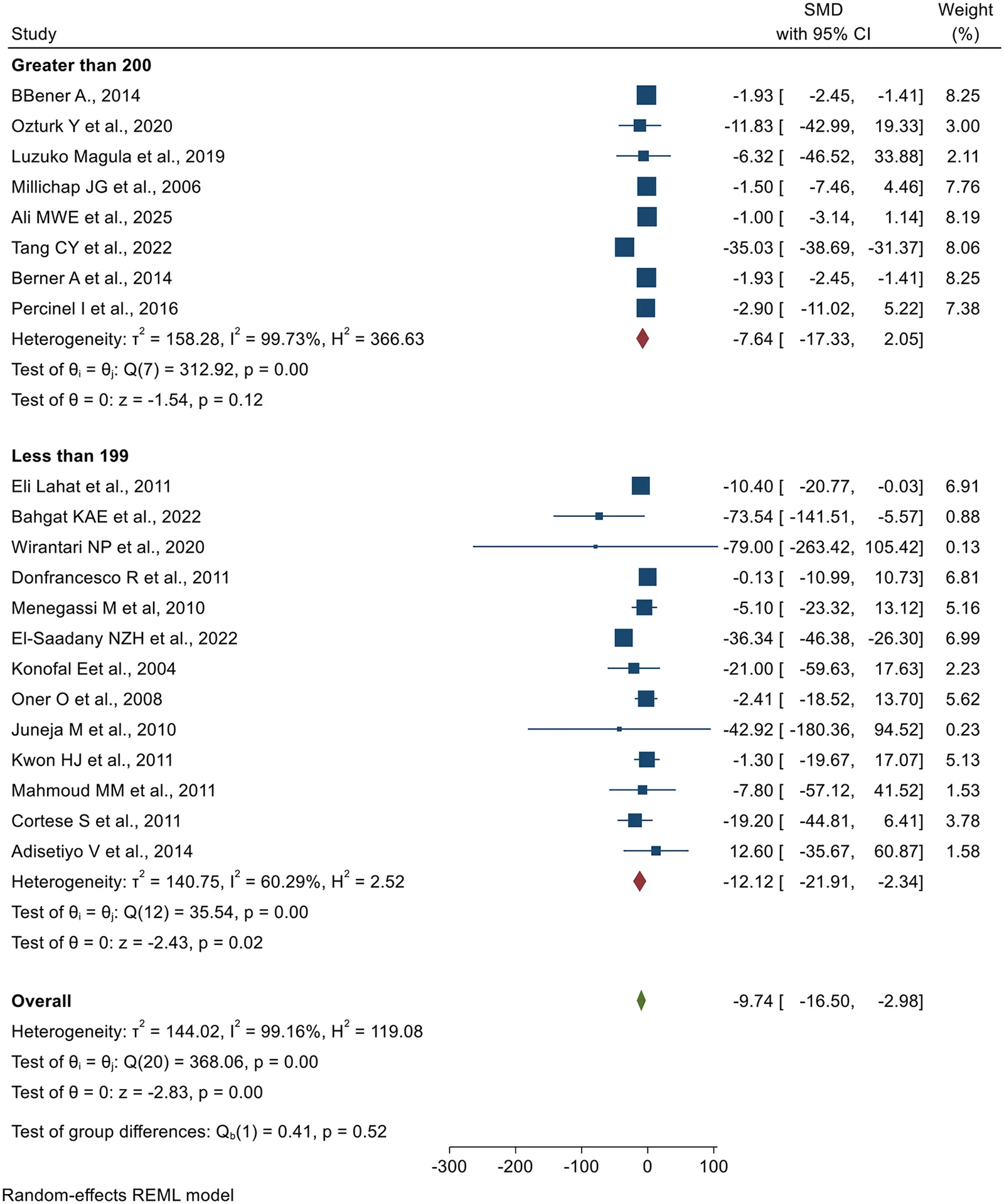
Forest plot showing sub-group analysis of the pooled MD of serum ferritin levels among children with ADHD and controls by sample size.

**Fig 8:**
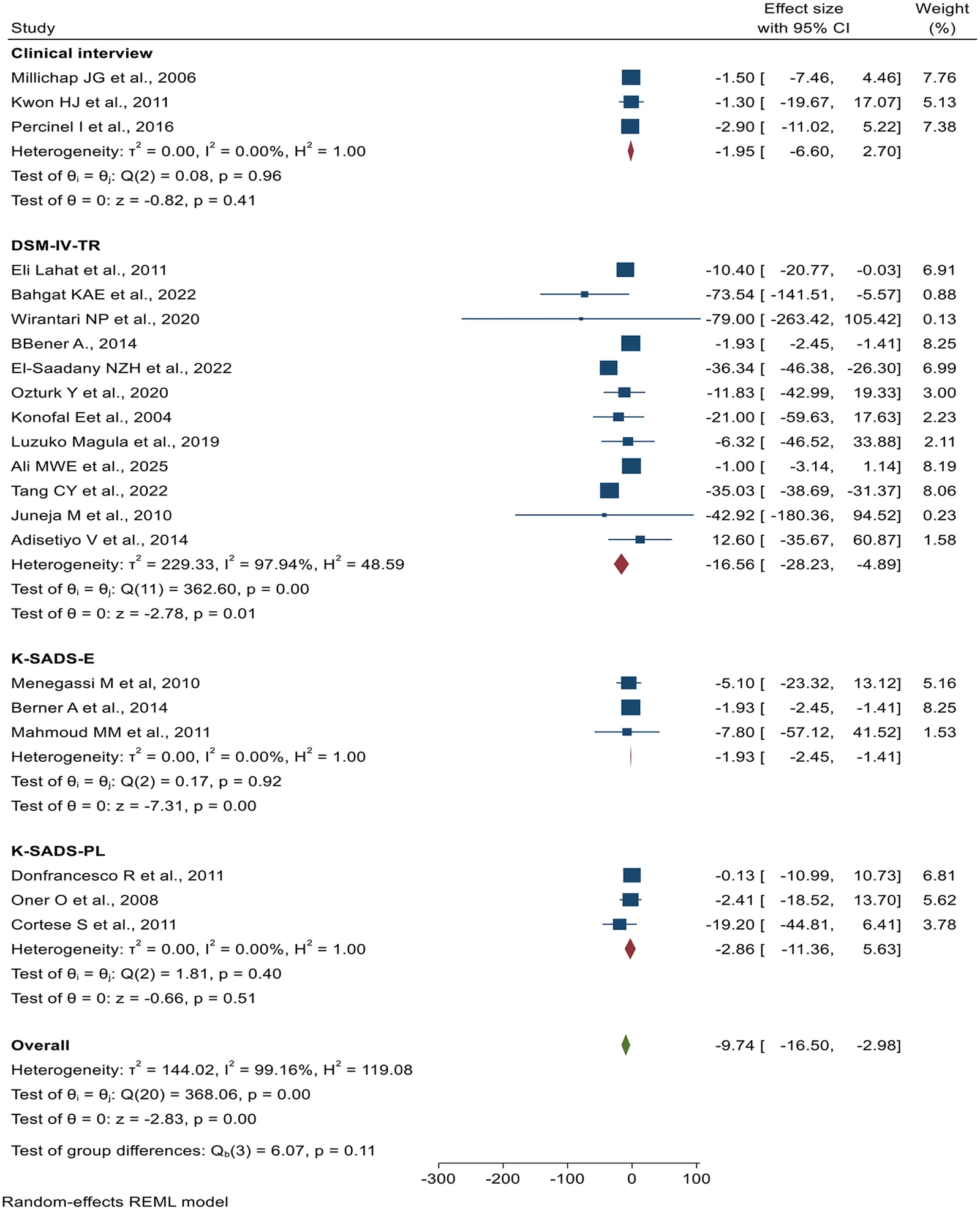
Forest plot showing sub-group analysis of the pooled MD of serum ferritin levels among children with ADHD and controls by ADHD diagnostic criteria.

### Publication bias

Included studies were assessed for potential publication bias visually by funnel plot and egger’s test statistics. Visual inspection of the funnel plots for included studies for serum ferritin revealed some asymmetry (Fig 9). However, Egger’s tests did not give significant evidence of publication bias among the included studies (P = 0.2987). (**Table 2**).

**Table 2:** Egger’s test for the Serum Ferritin in Children with Attention Deficit Hyperactivity Disorder (ADHD).

Std_Eff	Coef.	Std. Err.	z	P > z	[95% CI]	
slope	2.559617	9.513474	0.27	0.788	−16.08645	21.20568
bias	−0.45	0.437	−1.04	***0.2987***	−1.306	0.4066

***CI: Confidence interval; Std Eff: Standard effect; Coef: coefficient; Std. Err: Standard error.***

**Fig 9:**
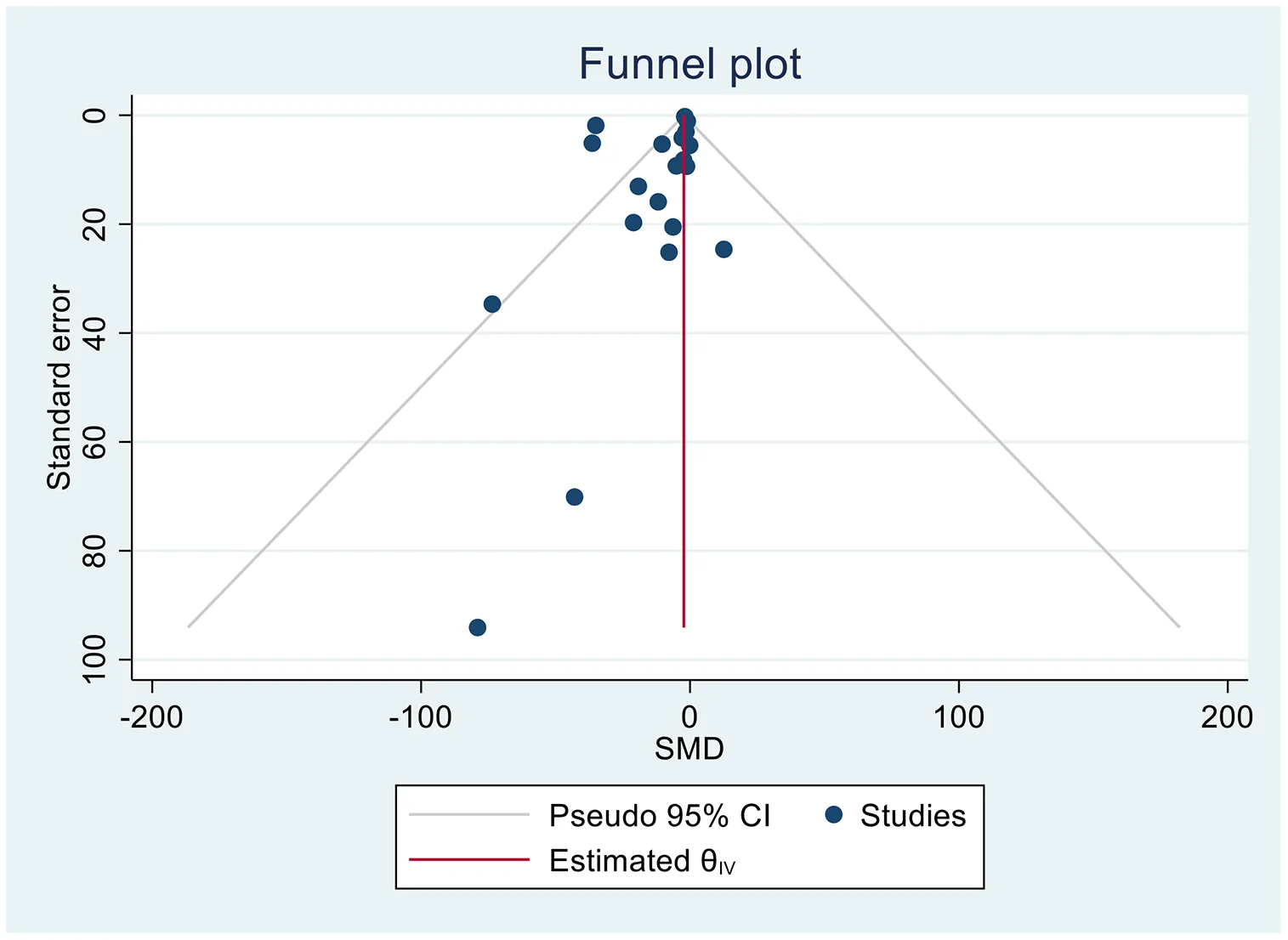
Funnel plot for the study Serum Ferritin in Children with Attention Deficit Hyperactivity Disorder (ADHD).

### Galbraith plot

The Galbraith plot shows that the majority of studies (19 out of 21) lie within the 95% confidence interval wedges, indicating that most studies are consistent with the overall pooled effect under a common-effect model. However, two studies fall outside the 95% CI boundaries, suggesting they are statistical outliers contributing to heterogeneity. Additionally, one study crosses the horizontal “no effect” line, indicating a null or non-significant association between serum ferritin levels and ADHD in that particular study. The presence of these three studies explains, at least in part, the observed statistical heterogeneity (I^2^) in the meta-analysis and supports the use of a random-effects model (Fig 10).

**Fig 10:**
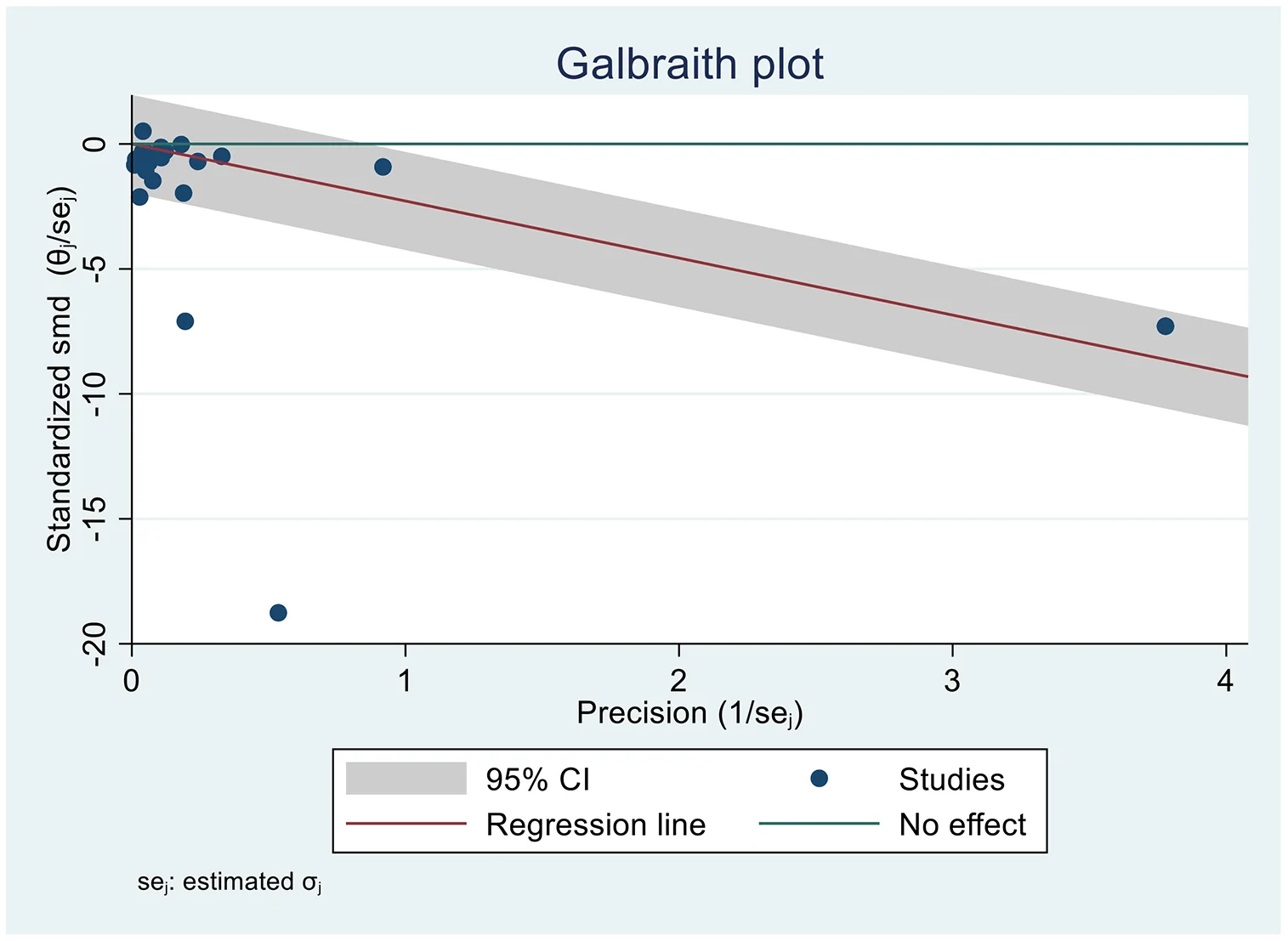
Galbraith plot for Heterogeneity assessment of the study Serum Ferritin in Children with Attention Deficit Hyperactivity Disorder (ADHD).

### Sensitivity analysis

A sensitivity analysis was conducted using a random-effects model to assess the influence of individual studies on the pooled estimate of SMD of serum ferritin in children with ADHD and controls. This was performed by sequentially omitting each study, and the results indicated that no single study had a substantial impact on the overall estimate (Fig 11).

**Fig 11:**
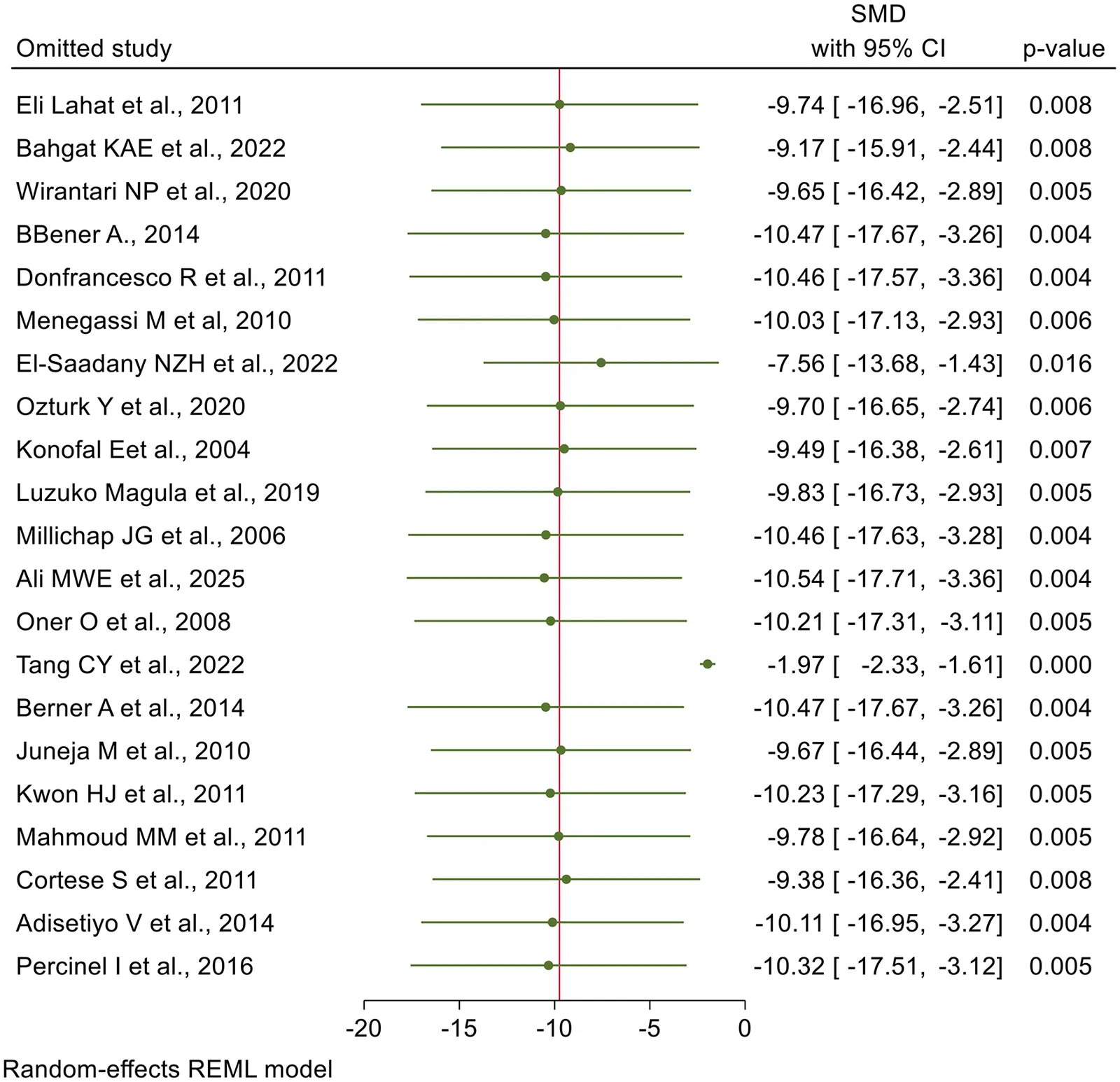
Sensitivity analysis of included studies on the pooled MD of Serum Ferritin in Children with Attention Deficit Hyperactivity Disorder (ADHD).

### Meta regression

To explore sources of heterogeneity among studies, we performed a meta-regression analysis by publication year, sample size, mean ferritin level of cases and mean ferritin level of controls. The result showed that mean ferritin level of cases and controls significantly influence the pooled MD estimate of serum ferritin among children with ADHD and healthy controls (**Table 3**).

**Table 3:** Meta-regression of serum ferritin in children with Attention Deficit Hyperactivity Disorder (ADHD).

meta_es	Coefficient	Std. err.	z	P > |z|	[95% conf. interval]	
Publication year	−0.8114602	0.5625756	−1.44	0.149	−1.914088	.2911678
Sample size	−0.0039784	0.0033672	−1.18	0.237	−.0105781	.0026213
Mean ferritin level of case	.9999999	.1758021	5.69	0.000	.6554341	1.344566
Mean ferritin level of controls	−.9999999	.0533763	−18.73	0.000	−1.104615	−.8953844
Constant	1627.752	1132.622	1.44	0.151	−592.146	3847.651

## Discussion

Iron deficiency has been shown to adversely affect cognitive, motor, social, and emotional development in children. At the neurobiological level, reduced brain iron concentrations are associated with disrupted cortical fiber conduction, alterations in serotonergic and dopaminergic neurotransmission, and impaired myelin formation [41]. Accordingly, this systematic review and meta-analysis aimed to determine the pooled mean difference in serum ferritin levels between children with ADHD and healthy controls. A total of 21 case-control and comparative cross-sectional studies, encompassing 8,591 participants (4,058 with ADHD), were included. The pooled analysis yielded a mean difference of −9.74 (95% CI: −16.50, −2.98), indicating significantly lower serum ferritin concentrations in the ADHD group compared with controls.

Iron, an important trace element, is implicated in many biological processes and plays a crucial role in the development of the brain [42].Iron deficiency influences the cognitive, motor, social and emotional functions in children [43]. It has been reported that decreased iron concentration in the brain is associated with alterations in the conduction of cortical fibers, changes in serotonergic and dopaminergic systems, and in the formation of myelin [44]. Serum ferritin, an intracellular protein that stores iron, is usually regarded as a reliable indicator of iron stores in body tissues, however, whether the serum ferritin is a good indicator of iron stores in the brain is debatable [45].The level of serum ferritin is affected by inflammation and food intake [46].Iron deficiency with or without anemia during childhood, especially in infancy, has a negative impact on cognition, behavior, and motor skills of children [47].Compared with serum iron, serum ferritin is a more sensitive marker which can be detected at the early stage of ID even without anemia.

In this meta-analysis, we found lower serum ferritin levels in patients with ADHD than in healthy controls. This finding is in agreement with a systematic review and meta-analysis conducted in 2017 [48], a study in Indonesia [36], Turkey [33], Egypt [27,28], China [49], and France [17]. Although the mechanism linking low iron to ADHD remains unclear, several hypotheses exist. Iron is a cofactor for tyrosine hydroxylase, the rate-limiting enzyme in dopamine synthesis, which is implicated in ADHD pathophysiology. Iron deficiency also reduces dopamine transporter density and activity, leading to increased extracellular dopamine and decreased striatal dopamine receptors, and may contribute to basal ganglia dysfunction. An imbalance between inhibitory gamma-aminobutyric acid (GABA) and excitatory neurotransmitters may play a role as well; lower GABA levels in ADHD patients could reduce brain iron concentration, particularly in the basal ganglia. Additionally, MRI studies have shown lower thalamic iron levels in children with ADHD, suggesting a potential etiological role [14,50–55]. Furthermore, Konofal et al.[56] and Sever et al.[57] Reported that after iron supplementation, the serum ferritin of ADHD patients increased significantly and ADHD symptoms improved. Based on these data it appears that iron supplementation may benefit ADHD.

On the other hand the findings of the current systematic review and meta-analysis is not consistent with findings from Italy [18], Brazil [38], Turkey ([34])and USA [20], that reports no significant difference in serum ferritin level between ADHD and controls. The possible variation could be attributed to difference in sample size, assay technique, feeding habit and socio-demographic differences. Additionally, discrepancies in ferritin level may be attributed to several factors, including population characteristics, sample collection methods, regional differences, sociodemographic influences, study design, and diagnostic approaches.

Given the substantial heterogeneity across studies (I^2^ = 99.16%, p-value = 0.00), subgroup analyses were conducted based on region, publication year, study design, and sample size. The results revealed that the highest pooled MD of serum ferritin was reported in the Africa (−19.28), followed by Asia (15.03) while the lowest was reported in North America (−1.42). This discrepancy may arise from differences in case severity, as ADHD symptom severity increases with greater serum ferritin depletion. Additional factors include variations in sample size, study design, and demographic location, which can influence feeding habits and food fortification.

When stratified by publication year, studies published before 2015 reported a pooled MD of −1.94, compared to −19.43 for those published after 2016. This significant variation indicates that older studies reported a lower pooled MD of serum ferritin level. This difference may be attributed to increased awareness increased case detection over time. Changes in environmental or lifestyle risk factors may also create the difference in cases and controls. Alternatively, variations in study design, sample selection, or diagnostic criteria between earlier and later research might explain the discrepancy, as could improvements in early diagnosis and case detection over time.

Subgroup analysis by study design revealed a pooled MD of −1.95 among case-control studies and −14.85 among comparative cross-sectional studies. The difference may be attributed to variations in sample size, socio-demographic characteristics, and lifestyle and feeding habits of the included participants.

Subgroup analysis by sample size revealed pooled MD of smaller sample sizes (<199 participants) yielded a higher pooled MD estimate of −12.12 (I^2^ = 60.29%, p = 0.00), whereas studies with larger sample sizes (≥200 participants) reported a lower MD of −7.64 (I^2^ = 99.73%, p = 0.00). The observed difference in pooled MD of studies with smaller and larger sample sizes may be attributed to several methodological and clinical factors. Smaller studies often focus on high-risk or specialized populations, which may inflate the estimates, whereas larger studies typically reflect more generalized populations, yielding lower but potentially more representative estimates. Additionally, smaller studies are more susceptible to random variability and selection bias due to limited sampling diversity, contributing to substantial heterogeneity. In contrast, larger studies benefit from broader participant inclusion and greater statistical power, enhancing reliability. These findings underscore the need for cautious interpretation of small-scale studies and highlight the value of large, population-based research for robust prevalence estimation. Furthermore, the subgroup analysis based on ADHD diagnostic criteria highlighted considerable variability across assessment tools. Mean differences ranged from −1.95 (95% CI: −6.60, 2.70) for clinical interviews to −16.56 (95% CI: −28.23, −4.89) for DSM-IV-TR criteria, with intermediate values observed for K-SADS-E (−1.96 [95% CI: −2.45, −1.41]) and K-SADS-PL (−2.86 [95% CI: −11.36, 5.63]). Of particular note, the DSM-IV-TR criteria produced the most pronounced mean difference, suggesting that diagnostic framework may substantially influence effect estimates and should be carefully considered when interpreting pooled findings.

Our meta-analysis shows that serum ferritin levels are lower in patients with ADHD than in healthy controls, which suggests that serum ferritin is correlated with ADHD. There is a need for more high-quality studies with larger sample sizes, assessed using the same assay techniques, and multiple indices of iron status to provide more conclusive results. The mechanisms leading to iron deficiency in ADHD, and the correlation between brain iron and peripheral iron levels also needs further research.

### Limitation and strength

This systematic review and meta-analysis has several limitations. First, the exclusion of non-English articles, necessitated by a lack of translation resources, may have introduced language bias. Second, the modest sample sizes of the included studies likely limited the statistical power of our analyses. Finally, the substantial heterogeneity observed across the studies warrants caution when interpreting the pooled results. Consequently, larger-scale studies are required to further clarify the relationship between serum ferritin levels and ADHD. Offsetting these limitations, however, is a key methodological strength: our comprehensive search strategy, which incorporated multiple databases and varied search approaches, ensures a broad and robust capture of the available evidence.

## Conclusion

This systematic review and meta-analysis yielded a pooled mean difference of –9.74 for serum ferritin levels between children with ADHD and healthy controls. In response to this marked deficiency, targeted interventions are warranted, including age-specific screening programs for at-risk children and enhanced iron supplementation protocols. Looking ahead, future research should prioritize longitudinal designs to establish the causal direction of the relationship between ferritin levels and ADHD, and to explore its potential association with symptom severity.

## Supporting information

S1 File Supplementary table. This file contains **S1 table** (The search strategy and number of articles retrieved from the searched databases for the review Serum Ferritin in Children with Attention Deficit Hyperactivity Disorder (ADHD): A Systematic Review and Meta-Analysis.).
(DOCX)

S2 File PRISMA 2020 Checklist. (DOCX)

## Abbreviations

ADHD: Attention Deficit Hyperactivity Disorder
CC: Case-control
CS: Cross-Sectional
DSM-IV-TR: Diagnostic and Statistical Manual of Mental Disorders, Fourth Edition, Text Revision
ID: Iron Deficiency
K-SADS-E: Kiddie Schedule for Affective Disorders and Schizophrenia–Epidemiological Version
K-SADS-PL: K-SADS–Present and Lifetime Version
MD: Mean Difference
MRI: Magnetic Resonance Imaging
PRISMA: Preferred Reporting Items for Systematic Review and Meta-Analysis
SMD: Standardized Mean Difference

## References

[1] Yackobovitch-Gavan M, Ben-Hefer D, Meyerovitch J. The association between iron deficiency anemia at infancy and attention deficit hyperactivity disorder. 2022. 10.1016/j.heliyon.2024.e33430 PMC11250855 39015811

[2] Black DW, Grant JE. DSM-5® guidebook: The essential companion to the diagnostic and statistical manual of mental disorders. American Psychiatric Pub. 2014.

[3] Biederman J. Attention-deficit/hyperactivity disorder: A selective overview. Biol Psychiatry. 2005;57(11):1215–20. doi: 10.1016/j.biopsych.2004.10.020 15949990

[4] Luo Y, Weibman D, Halperin JM, Li X. A review of heterogeneity in attention deficit/hyperactivity disorder (ADHD). Frontiers in Human Neuroscience. 2019;13:42. 10.3389/fnhum.2019.00042 PMC6378275 30804772

[5] Thomas R, Sanders S, Doust J, Beller E, Glasziou P. Prevalence of attention-deficit/hyperactivity disorder: a systematic review and meta-analysis. Pediatrics. 2015;135(4):e994-1001. doi: 10.1542/peds.2014-3482 25733754

[6] Chang Y-L, Hung S-H, Ling W, Lin H-C, Li H-C, Chung S-D. Association between ischemic stroke and iron-deficiency anemia: a population-based study. PLoS One. 2013;8(12):e82952. doi: 10.1371/journal.pone.0082952 24349404 PMC3857285

[7] Greenblatt JM, Delane DD. Micronutrient deficiencies in ADHD: A global research consensus. Journal of Orthomolecular Medicine. 2017;32(6).

[8] Biederman J, Faraone S. Attention—deficit hyperactivity disorder. Lancet. 2005;366(9481):237–48. 10.1016/S0140-6736(05)66915-2 16023516

[9] Sinn N. Nutritional and dietary influences on attention deficit hyperactivity disorder. Nutr Rev. 2008;66(10):558–68. doi: 10.1111/j.1753-4887.2008.00107.x 18826452

[10] Swanson JM, Kinsbourne M, Nigg J, Lanphear B, Stefanatos GA, Volkow N, et al. Etiologic subtypes of attention-deficit/hyperactivity disorder: brain imaging, molecular genetic and environmental factors and the dopamine hypothesis. Neuropsychol Rev. 2007;17(1):39–59. doi: 10.1007/s11065-007-9019-9 17318414

[11] Faraone SV, Perlis RH, Doyle AE, Smoller JW, Goralnick JJ, Holmgren MA, et al. Molecular genetics of attention-deficit/hyperactivity disorder. Biol Psychiatry. 2005;57(11):1313–23. doi: 10.1016/j.biopsych.2004.11.024 15950004

[12] Rucklidge JJ, Johnstone J, Kaplan BJ. Nutrient supplementation approaches in the treatment of ADHD. Expert Rev Neurother. 2009;9(4):461–76. doi: 10.1586/ern.09.7 19344299

[13] Pelsser LMJ, Frankena K, Toorman J, Savelkoul HFJ, Pereira RR, Buitelaar JK. A randomised controlled trial into the effects of food on ADHD. Eur Child Adolesc Psychiatry. 2009;18(1):12–9. doi: 10.1007/s00787-008-0695-7 18431534

[14] Cortese S, Azoulay R, Castellanos FX, Chalard F, Lecendreux M, Chechin D, et al. Brain iron levels in attention-deficit/hyperactivity disorder: A pilot MRI study. World J Biol Psychiatry. 2012;13(3):223–31. doi: 10.3109/15622975.2011.570376 21585274

[15] Juneja M, Jain R, Singh V, Mallika V. Iron deficiency in Indian children with attention deficit hyperactivity disorder. Indian Pediatr. 2010;47(11):955–8. doi: 10.1007/s13312-010-0160-9 20453262

[16] Adisetiyo V, Jensen JH, Tabesh A, Deardorff RL, Fieremans E, Di Martino A, et al. Multimodal MR imaging of brain iron in attention deficit hyperactivity disorder: a noninvasive biomarker that responds to psychostimulant treatment?. Radiology. 2014;272(2):524–32. doi: 10.1148/radiol.14140047 24937545 PMC4263268

[17] Konofal E, Lecendreux M, Arnulf I, Mouren M-C. Iron deficiency in children with attention-deficit/hyperactivity disorder. Arch Pediatr Adolesc Med. 2004;158(12):1113–5. doi: 10.1001/archpedi.158.12.1113 15583094

[18] Donfrancesco R, Parisi P, Vanacore N, Martines F, Sargentini V, Cortese S. Iron and ADHD: Time to move beyond serum ferritin levels. J Atten Disord. 2013;17(4):347–57. doi: 10.1177/1087054711430712 22290693

[19] Oner O, Alkar OY, Oner P. Relation of ferritin levels with symptom ratings and cognitive performance in children with attention deficit-hyperactivity disorder. Pediatr Int. 2008;50(1):40–4. doi: 10.1111/j.1442-200X.2007.02496.x 18279203 PMC3465160

[20] Millichap JG, Yee MM, Davidson SI. Serum ferritin in children with attention-deficit hyperactivity disorder. Pediatr Neurol. 2006;34(3):200–3. doi: 10.1016/j.pediatrneurol.2005.09.001 16504789

[21] Page MJ, McKenzie JE, Bossuyt PM, Boutron I, Hoffmann TC, Mulrow CD, et al. The PRISMA 2020 statement: an updated guideline for reporting systematic reviews. Revista panamericana de salud publica. 2022;46:e112. 10.26633/RPSP.2022.112 PMC9798848 36601438

[22] Hoy D, Brooks P, Woolf A, Blyth F, March L, Bain C, et al. Assessing risk of bias in prevalence studies: modification of an existing tool and evidence of interrater agreement. J Clin Epidemiol. 2012;65(9):934–9. doi: 10.1016/j.jclinepi.2011.11.014 22742910

[23] French Intensive Care Society. International congress - Réanimation 2016. Ann Intensive Care. 2016;6(Suppl 1):50. 10.1186/s13613-016-0114-z PMC4912513 27316915

[24] Higgins JPT, Thompson SG. Quantifying heterogeneity in a meta-analysis. Stat Med. 2002;21(11):1539–58. doi: 10.1002/sim.1186 12111919

[25] Shi X, Nie C, Shi S, Wang T, Yang H, Zhou Y. Effect comparison between Egger’s test and Begg’s test in publication bias diagnosis in meta-analyses: evidence from a pilot survey. Int J Res Stud Biosci. 2017;5(5):14–20.

[26] Page MJ, McKenzie JE, Bossuyt PM, Boutron I, Hoffmann TC, Mulrow CD, et al. The PRISMA 2020 statement: an updated guideline for reporting systematic reviews. BMJ. 2021;372:n71. doi: 10.1136/bmj.n71 33782057 PMC8005924

[27] Bahgat KAE, Nasr HM, El-Sayed SK. Effect of iron deficiency on behavior and cognition in children. Int J Health Sci. 2022;:998–1011. doi: 10.53730/ijhs.v6ns7.11490

[28] El-Saadany NZH, Abdel-Fattah MF, Siam AG, Abdel-Fattah NR. Relationship between serum iron level and zinc level with attention deficit hyperactivity disorder in children. The Egyptian J Hospital Med. 2022;87(1):2106–12. doi: 10.21608/ejhm.2022.232828

[29] Ali MWE, Mahrous DM, Ebrahim NHN, Marey H. Iron profile in children with ADHD. Minia J Medical Res. 2025;36(2):97–105. doi: 10.21608/mjmr.2025.375506.1932

[30] Mahmoud MM, El-Mazary A-AM, Maher RM, Saber MM. Zinc, ferritin, magnesium and copper in a group of Egyptian children with attention deficit hyperactivity disorder. Ital J Pediatr. 2011;37:60. doi: 10.1186/1824-7288-37-60 22206662 PMC3268715

[31] Lahat E, Heyman E, Livne A, Goldman M, Berkovitch M, Zachor D. Iron deficiency in children with attention deficit hyperactivity disorder. Isr Med Assoc J. 2011;13(9):530–3. 21991711

[32] Tang C-Y, Wen F. Serum ferritin levels in children with attention deficit hyperactivity disorder and tic disorder. World J Clin Cases. 2022;10(22):7749–59. doi: 10.12998/wjcc.v10.i22.7749 36158507 PMC9372851

[33] Bener A. Higher prevalence of iron deficiency as strong predictor of attention deficit hyperactivity disorder in children. European Psychiatry. 2015;30:691. doi: 10.1016/s0924-9338(15)30547-2 PMC4212392 25364604

[34] Öztürk Y, Topal Z, Demir N, Tufan AE. Iron and Ferritin levels of children and adolescents with attention deficit hyperactivity disorder and attention deficit hyperactivity disorder-not otherwise specified. jpr. 2020;7(3):216–22. doi: 10.4274/jpr.galenos.2019.57625

[35] Magula L, Moxley K, Lachman A. Iron deficiency in South African children and adolescents with attention deficit hyperactivity disorder. J Child Adolesc Ment Health. 2019;31(2):85–92. doi: 10.2989/17280583.2019.1637345 31339453

[36] Wirantari NP, Windiani IGAT, Wati DK, Sidiartha IGL, Karyana IPG, Gustawan IW. Association of ferritin level with attention deficit hyperactivity disorder in children. Int J Res Med Sci. 2020;8(5):1679. doi: 10.18203/2320-6012.ijrms20201908

[37] Bener A, Kamal M, Bener H, Bhugra D. Higher prevalence of iron deficiency as strong predictor of attention deficit hyperactivity disorder in children. Ann Med Health Sci Res. 2014;4(Suppl 3):S291-7. doi: 10.4103/2141-9248.141974 25364604 PMC4212392

[38] Menegassi M, Mello ED de, Guimarães LR, Matte BC, Driemeier F, Pedroso GL, et al. Food intake and serum levels of iron in children and adolescents with attention-deficit/hyperactivity disorder. Braz J Psychiatry. 2010;32(2):132–8. doi: 10.1590/s1516-44462009005000008 19838594

[39] Percinel I, Yazici KU, Ustundag B. Iron deficiency parameters in children and adolescents with attention-deficit/hyperactivity disorder. Child Psychiatry Hum Dev. 2016;47(2):259–69. doi: 10.1007/s10578-015-0562-y 26092605

[40] Kwon HJ, Lim MH, Ha M, Kim EJ, Yoo SJ, Kim JW, et al. Transferrin in Korean children with attention deficit hyperactivity disorder. Psychiatry Investig. 2011;8(4):366–71. doi: 10.4306/pi.2011.8.4.366 22216048 PMC3246146

[41] Ben-Shachar D, Livne E, Spanier I, Zuk R, Youdim MB. Iron modulates neuroleptic-induced effects related to the dopaminergic system. Isr J Med Sci. 1993;29(9):587–92. 7901181

[42] Beard JL. Why iron deficiency is important in infant development. J Nutr. 2008;138(12):2534–6. doi: 10.1093/jn/138.12.2534 19022985 PMC3415871

[43] Lozoff B, Beard J, Connor J, Barbara F, Georgieff M, Schallert T. Long-lasting neural and behavioral effects of iron deficiency in infancy. Nutr Rev. 2006;64(5 Pt 2):S34-43; discussion S72-91. doi: 10.1301/nr.2006.may.s34-s43 16770951 PMC1540447

[44] Beard J. Iron deficiency alters brain development and functioning. J Nutr. 2003;133(5 Suppl 1):1468S-72S. doi: 10.1093/jn/133.5.1468S 12730445

[45] Oner O, Oner P, Bozkurt OH, Odabas E, Keser N, Karadag H, et al. Effects of zinc and ferritin levels on parent and teacher reported symptom scores in attention deficit hyperactivity disorder. Child Psychiatry Hum Dev. 2010;41(4):441–7. doi: 10.1007/s10578-010-0178-1 20238159 PMC3399584

[46] Thurnham DI, Northrop-Clewes CA. Inflammation and biomarkers of micronutrient status. Curr Opin Clin Nutr Metab Care. 2016;19(6):458–63. doi: 10.1097/MCO.0000000000000323 27583708

[47] Jáuregui-Lobera I. Iron deficiency and cognitive functions. Neuropsychiatr Dis Treat. 2014;10:2087–95. doi: 10.2147/NDT.S72491 25419131 PMC4235202

[48] Wang Y, Huang L, Zhang L, Qu Y, Mu D. Iron status in attention-Deficit/Hyperactivity Disorder: A Systematic Review and Meta-Analysis. PLoS One. 2017;12(1):e0169145. doi: 10.1371/journal.pone.0169145 28046016 PMC5207676

[49] Cheung YT, Chan DFY, Lee CK, Tsoi WC, Lau CW, Leung JNS, et al. Impact of iron deficiency on attention among school-aged adolescents in Hong Kong. Hong Kong Med J. 2025;31(2):139–47. doi: 10.12809/hkmj2310950 40201988

[50] Erikson KM, Jones BC, Beard JL. Iron deficiency alters dopamine transporter functioning in rat striatum. J Nutr. 2000;130(11):2831–7. doi: 10.1093/jn/130.11.2831 11053528

[51] Lozoff B. Early iron deficiency has brain and behavior effects consistent with dopaminergic dysfunction. J Nutr. 2011;141(4):740S-746S. doi: 10.3945/jn.110.131169 21346104 PMC3056585

[52] Youdim MB, Ben-Shachar D, Yehuda S. Putative biological mechanisms of the effect of iron deficiency on brain biochemistry and behavior. Am J Clin Nutr. 1989;50(3 Suppl):607–15; discussion 615-7. doi: 10.1093/ajcn/50.3.607 2773840

[53] Gallo EF, Posner J. Moving towards causality in attention-deficit hyperactivity disorder: overview of neural and genetic mechanisms. Lancet Psychiatry. 2016;3(6):555–67. doi: 10.1016/S2215-0366(16)00096-1 27183902 PMC4893880

[54] Edden RAE, Crocetti D, Zhu H, Gilbert DL, Mostofsky SH. Reduced GABA concentration in attention-deficit/hyperactivity disorder. Arch Gen Psychiatry. 2012;69(7):750–3. doi: 10.1001/archgenpsychiatry.2011.2280 22752239 PMC3970207

[55] Hill JM. Iron concentration reduced in ventral pallidum, globus pallidus, and substantia nigra by GABA-transaminase inhibitor, gamma-vinyl GABA. Brain Res. 1985;342(1):18–25. doi: 10.1016/0006-8993(85)91348-4 4041811

[56] Konofal E, Lecendreux M, Deron J, Marchand M, Cortese S, Zaïm M, et al. Effects of iron supplementation on attention deficit hyperactivity disorder in children. Pediatr Neurol. 2008;38(1):20–6. doi: 10.1016/j.pediatrneurol.2007.08.014 18054688

[57] Sever Y, Ashkenazi A, Tyano S, Weizman A. Iron treatment in children with attention deficit hyperactivity disorder. A preliminary report. Neuropsychobiology. 1997;35(4):178–80. doi: 10.1159/000119341 9246217

