# Supplementary material for: Serum ferritin in children with Attention Deficit Hyperactivity Disorder (ADHD): A systematic review and meta-analysis
Source: PLoS One. 2026 Oct 5;21(10):e0359790. doi: 10.1371/journal.pone.0359790 (PMC13637864; doi:10.1371/journal.pone.0359790)
Supplement: Supplementary file 1 [file pone.0359790.s004.pdf]

Authors point by point Response to Reviewers:

Date: September 07, 2026

Manuscript ID: PONE-D-26-33165

Title: Serum Ferritin in Children with Attention Deficit Hyperactivity Disorder (ADHD): A Systematic Review and Meta-Analysis

Dear Dr. Chen, (Chief Academic Editor)

Thank you for the opportunity to revise our manuscript. We also sincerely thank the editor and the reviewers for their time, effort, and constructive feedback. We have addressed all comments point-by-point and believe the manuscript has been substantially improved as a result.

Below, editor and reviewer comments are in "bold" with our responses in plain text. All page numbers refer to the "Revised Manuscript with Track Changes" file. Furthermore, for language and typographical corrections, we have thoroughly revised the entire manuscript. These corrections have been made throughout the document and are not highlighted in color, as they are integrated comprehensively across the text. We remain fully open to any additional suggestions, clarifications, or amendments you wish to recommend, and we would be happy to address them promptly.

Editor comment #1: **Please ensure that your manuscript meets PLOS ONE's style requirements, including those for file naming.**

Author Response: We have reformatted the manuscript accordingly. All files now follow PLOS ONE naming conventions.

Editor comment #2: **Please include captions for your Supporting Information files at the end of your manuscript, and update any in-text citations to match accordingly.**

Author Response: We are grateful for this valuable comment. In response, we have added full captions for supporting information item, as recommended (revised manuscript, page 35).

Editor comment #3: **If the reviewer comments include a recommendation to cite specific previously published works, please review and evaluate these publications to determine**

whether they are relevant and should be cited. There is no requirement to cite these works unless the editor has indicated otherwise.

Author Response: We appreciate the editor's insight. To clarify: no reviewer asked us to cite any specific previously published work.

Review Comments to the Author

Reviewer 1 comments

Review 1 comment #1: The author should report their PICOS

Author Response: We sincerely appreciate the reviewer's suggestion to report the PICOS in our review. As per PRISMA guidelines, we have now explicitly detailed the PICOS framework used for our eligibility criteria. **Review question**: children (**population**) with ADHD (**exposure**) had lower levels of mean serum ferritin (**outcome**) than healthy controls (**comparison**)?

Population: Children

Intervention (exposure): Diagnosis of ADHD (presence of ADHD could affect serum ferritin level or not)

Comparison: Healthy control (children without ADHD)

Outcome: Mean serum ferritin level (comparing the difference in mean serum ferritin level between two groups)

Review 1 comment #2: Regarding the presentation order of the Results section, the first paragraph describing the search results and the second paragraph presenting the study characteristics are appropriate. The third paragraph should report the methodological quality of the included studies, including the risk-of-bias assessment. The fourth paragraph should then present the main outcome, corresponding to Figure 5. Subsequent paragraphs may address publication bias, sensitivity analyses, meta-regression, and other supplementary analyses.

Author Response: We sincerely thank the reviewer for this thoughtful and constructive suggestion regarding the logical flow of our Results section. We fully agree that a structured, stepwise presentation moving from search results to study characteristics, then to methodological quality,

followed by the primary outcome, and finally to supplementary analyses greatly enhances the readability and scientific rigor of the manuscript.

These revisions have been implemented throughout pages 10–26 of the revised manuscript. All changes have been made using track changes for easy identification.

Review 1 comment #3: For laboratory measures with established reference ranges, such as ferritin levels, reporting the mean difference (MD) rather than only the standardized mean difference (SMD) would allow readers to better appreciate the actual magnitude of the difference between the ADHD and control groups in clinically interpretable units. The SMD may be retained to facilitate comparison across studies; however, we recommend additionally reporting the pooled MD whenever the original measurements are expressed in the same unit.

Author Response: We sincerely thank the reviewer for this excellent insight. In response to this comment, we apologize for this typo error, the forest plots analyzed in the document are pooled mean differences rather than the standard mean difference. That is truly a typo error, for your assurance we can attach the excel data as a supportive document based on reasonable requests. Now we calculated and incorporated the pooled SMD alongside the mean difference. This additional analysis is now presented in the revised Results section (pages 17).

Review 1 comment #4: Because "iron deficiency" was included in the search terms, and because ferritin levels in the ADHD groups of several studies appeared particularly low, possibly reaching the threshold for iron deficiency—an analysis of the association between iron deficiency status (yes/no) and ADHD status (yes/no), expressed as an odds ratio, would also be highly informative and clinically meaningful.

More importantly, such an analysis could help clarify whether ADHD is associated primarily with ferritin levels that fall substantially below the normal range and meet the definition of iron deficiency, or whether ferritin levels that remain within the normal range but are relatively low are also associated with an increased risk of ADHD.

Author Response: we appreciate the reviewer insight. In response we are here analyze, whether there is difference in ferritin level between ADHD and normal children. Of course, we plan to conduct a systematic review and meta-analysis on prevalence and associated factors of iron

deficiency and iron deficiency anemia among children with ADHD. In light of this recommendation, we have now conducted additional analyses to compare ferritin levels between children with ADHD and healthy controls, as presented in our revised manuscript. Furthermore, we plan to extend this line of inquiry through a separate systematic review and meta-analysis specifically focused on the prevalence and associated factors of iron deficiency and iron deficiency anemia among children with ADHD. We believe this will address the broader clinical question raised by the reviewer and contribute to a more nuanced understanding of the iron-ADHD relationship.

Review 1 comment #5: I noticed that the units for ferritin levels are not reported in Table 1. Moreover, the ferritin values vary substantially across studies, raising the possibility that different measurement units were used. Please specify the unit used in each study and convert all ferritin values to a common unit to ensure consistency and comparability.

Author Response: We appreciate the reviewer's attention to this detail. The unit for ferritin levels (ng/mL) has now been clearly indicated in the Results section table 1, (pages10 -13).

Review 1 comment #6: The subgroup analyses showed substantial differences between studies published before versus after 2015/2016, as well as between studies conducted in Asia/Africa and those conducted in North America/Europe. As discussed in paragraphs 4–6 of the Discussion, these differences may reflect variations in ADHD severity, environmental factors, or measurement methods.

To further examine these hypotheses, I suggest conducting the following additional analyses:

1. A meta-regression using the pooled ferritin level across the case and control groups as a moderator.

2. A meta-regression using ADHD severity as a moderator.

3. A subgroup analysis according to the diagnostic methods or assessment tools used to identify ADHD.

These analyses could help determine whether the observed heterogeneity is attributable to baseline ferritin status, ADHD severity, or differences in diagnostic and measurement

approaches, thereby providing empirical support for the explanations proposed in the Discussion.

Author Response: We thank the reviewer for this constructive suggestion. In response, we have conducted meta-regression analyses incorporating the pooled ferritin level across the case and control groups as a moderator, to explore potential sources of heterogeneity (page 24). Additionally, we performed subgroup analyses based on the ADHD diagnostic criteria used to identify ADHD. These additional analyses have been incorporated into the revised manuscript (pages [15 and 22]), and the findings are discussed accordingly. On the other hand, meta-regression analyses according to ADHD severity were not feasible, because none of the included studies reported adequate data on the severity of ADHD symptoms.

Reviewer 2 comments

Review 2 comment #1: This systematic review and meta-analysis investigated the association between attention-deficit/hyperactivity disorder (ADHD) and serum ferritin levels. The study was conducted in accordance with the Preferred Reporting Items for Systematic Reviews and Meta-Analyses (PRISMA) guidelines. A total of 7,643 articles were initially identified, and 21 studies, comprising 4,058 children with ADHD and 4,533 healthy controls, were ultimately included in the meta-analysis. The pooled results indicated that lower serum ferritin levels were significantly associated with ADHD in children (pooled SMD: −9.74), suggesting that screening for serum ferritin levels and appropriate iron supplementation may be considered in this population.

Overall, this review was conducted rigorously, and the manuscript is well organized and clearly written. I have one minor comment. Several relevant studies appear not to have been included in the meta-analysis. The authors should clarify the reasons for their exclusion and explain whether these studies met the predefined eligibility criteria.

Author Response: We thank the reviewer for this important observation. To enhance transparency regarding our study selection process, we have now provided a detailed description of our eligibility criteria in the Methods section (page [6]). Additionally, the PRISMA flow diagram (Figure 1) has been carefully reviewed to ensure that the inclusion and exclusion of articles are

clearly and comprehensively presented, including the specific reasons for exclusions at each stage. We believe these revisions adequately address the reviewer's concern.
