# Supplementary material for: Serum ferritin in children with Attention Deficit Hyperactivity Disorder (ADHD): A systematic review and meta-analysis
Source: PLoS One. 2026 Oct 5;21(10):e0359790. doi: 10.1371/journal.pone.0359790 (PMC13637864; doi:10.1371/journal.pone.0359790)
Supplement: Supplementary file 2 [file pone.0359790.s001.docx]

Serum Ferritin in Children with Attention Deficit Hyperactivity Disorder (ADHD): A Systematic Review and Meta-Analysis

Supplementary file

**S1 Table: The search strategy and number of articles retrieved from the searched databases for the review Serum Ferritin in Children with Attention Deficit Hyperactivity Disorder (ADHD)**

| Database | Searching strengs | Number of paper identified |
| --- | --- | --- |
| pubmed | (((((((((((((serum ferritin) OR (ferritin level)) OR (serum iron)) OR (iron deficiency)) OR (ID)) OR (iron deficiency anemia)) OR (IDA)) AND (children)) AND (Attention Deficit Hyperactivity Disorder)) OR (ADHD)) OR (Attention Deficit Disorder)) OR (ADD)) OR (Hyperkinetic Disorder)) OR (Hyperkinesis) Filters: Free full text, Clinical Study, Clinical Trial, Clinical Trial, Phase I, Clinical Trial, Phase II, Controlled Clinical Trial, Observational Study, Randomized Controlled Trial, English, Humans, Child: birth-18 years, from 2000 - 2026 | 2,046 |
| ScienceDirect | serum ferritin OR serum iron OR Iron deficiency OR iron deficiency anemia among children with attention deficit disorder hyperactivity OR ADHD OR attention deficit disorder OR ADD, from 2000 - 2026 | 512 |
| Scopus | Serum ferritin* OR serum iron* OR Iron deficiency* AND children* AND ADHD* OR attention deficit hyperactivity disorder* OR attention deficit disorder* OR ADD* | 452 |
| Google scholar | Serum ferritin level among children with attention deficit hyperactivity disorder with, ADHD, 2000-2026 | 4621 |
| Other /manual |  | 12 |
