# Supplementary material for: Serum ferritin in children with Attention Deficit Hyperactivity Disorder (ADHD): A systematic review and meta-analysis
Source: PLoS One. 2026 Oct 5;21(10):e0359790. doi: 10.1371/journal.pone.0359790 (PMC13637864; doi:10.1371/journal.pone.0359790)
Supplement: Peer review history

# Peer review: Decision Letter 0

**DOI:** 10.1371/journal.pone.0359790.r001

**Authors:** Mu-Hong Chen

7 Aug 2026

Dear Dr. Gedfie,

Thank you for submitting your manuscript to PLOS ONE. After careful consideration, we feel that it has merit but does not fully meet PLOS ONE’s publication criteria as it currently stands. Therefore, we invite you to submit a revised version of the manuscript that addresses the points raised during the review process.

Please submit your revised manuscript by Oct 06 2026 11:59PM. If you will need more time than this to complete your revisions, please reply to this message or contact the journal office at plosone@plos.org. When you're ready to submit your revision, log on to https://www.editorialmanager.com/pone/ and select the 'Submissions Needing Revision' folder to locate your manuscript file.

- A letter that responds to each point raised by the academic editor and reviewer(s). You should upload this letter as a separate file labeled 'Response to Reviewers'.
- A marked-up copy of your manuscript that highlights changes made to the original version. You should upload this as a separate file labeled 'Revised Manuscript with Track Changes'.
- An unmarked version of your revised paper without tracked changes. You should upload this as a separate file labeled 'Manuscript'.

If you would like to make changes to your financial disclosure, please include your updated statement in your cover letter. Guidelines for resubmitting your figure files are available below the reviewer comments at the end of this letter.

If applicable, we recommend that you deposit your laboratory protocols in protocols.io to enhance the reproducibility of your results. Protocols.io assigns your protocol its own identifier (DOI) so that it can be cited independently in the future. For instructions see: https://journals.plos.org/plosone/s/submission-guidelines#loc-laboratory-protocols. Additionally, PLOS ONE offers an option for publishing peer-reviewed Lab Protocol articles, which describe protocols hosted on protocols.io. Read more information on sharing protocols at https://plos.org/protocols?utm_medium=editorial-email&utm_source=authorletters&utm_campaign=protocols.

As the corresponding author, your ORCID iD is verified in the submission system and will appear in the published article. PLOS supports the use of ORCID, and we encourage all coauthors to register for an ORCID iD and use it as well. Please encourage your coauthors to verify their ORCID iD within the submission system before final acceptance, as unverified ORCID iDs will not appear in the published article. *Only*  the individual author can complete the verification step; PLOS staff *cannot*  verify ORCID iDs on behalf of authors.

We look forward to receiving your revised manuscript.

Kind regards,

Mu-Hong Chen, M.D., Ph.D.

Academic Editor

PLOS One

Journal Requirements:

When submitting your revision, we need you to address these additional requirements.

1. Please ensure that your manuscript meets PLOS ONE's style requirements, including those for file naming. The PLOS ONE style templates can be found at

https://journals.plos.org/plosone/s/file?id=wjVg/PLOSOne_formatting_sample_main_body.pdf and

https://journals.plos.org/plosone/s/file?id=ba62/PLOSOne_formatting_sample_title_authors_affiliations.pdf

2. Please include captions for your Supporting Information files at the end of your manuscript, and update any in-text citations to match accordingly. Please see our Supporting Information guidelines for more information: http://journals.plos.org/plosone/s/supporting-information.

3. If the reviewer comments include a recommendation to cite specific previously published works, please review and evaluate these publications to determine whether they are relevant and should be cited. There is no requirement to cite these works unless the editor has indicated otherwise.

[Note: HTML markup is below. Please do not edit.]

Reviewers' comments:

Reviewer's Responses to Questions

**Comments to the Author**

1. Is the manuscript technically sound, and do the data support the conclusions?

Reviewer #1: Yes

Reviewer #2: Yes

**********

2. Has the statistical analysis been performed appropriately and rigorously?

Reviewer #1: Yes

Reviewer #2: No

**********

3. Have the authors made all data underlying the findings in their manuscript fully available?

The PLOS Data policy

Reviewer #1: Yes

Reviewer #2: Yes

**********

4. Is the manuscript presented in an intelligible fashion and written in standard English?

Reviewer #1: Yes

Reviewer #2: Yes

**********

Reviewer #1: 1. The author should report their PICOS

2. Regarding the presentation order of the Results section, the first paragraph describing the search results and the second paragraph presenting the study characteristics are appropriate. The third paragraph should report the methodological quality of the included studies, including the risk-of-bias assessment. The fourth paragraph should then present the main outcome, corresponding to Figure 5. Subsequent paragraphs may address publication bias, sensitivity analyses, meta-regression, and other supplementary analyses.

3. For laboratory measures with established reference ranges, such as ferritin levels, reporting the mean difference (MD) rather than only the standardized mean difference (SMD) would allow readers to better appreciate the actual magnitude of the difference between the ADHD and control groups in clinically interpretable units. The SMD may be retained to facilitate comparison across studies; however, we recommend additionally reporting the pooled MD whenever the original measurements are expressed in the same unit.

4. Because “iron deficiency” was included in the search terms, and because ferritin levels in the ADHD groups of several studies appeared particularly low—possibly reaching the threshold for iron deficiency—an analysis of the association between iron deficiency status (yes/no) and ADHD status (yes/no), expressed as an odds ratio, would also be highly informative and clinically meaningful.

More importantly, such an analysis could help clarify whether ADHD is associated primarily with ferritin levels that fall substantially below the normal range and meet the definition of iron deficiency, or whether ferritin levels that remain within the normal range but are relatively low are also associated with an increased risk of ADHD.

5. I noticed that the units for ferritin levels are not reported in Table 1. Moreover, the ferritin values vary substantially across studies, raising the possibility that different measurement units were used. Please specify the unit used in each study and convert all ferritin values to a common unit to ensure consistency and comparability.

6. The subgroup analyses showed substantial differences between studies published before versus after 2015/2016, as well as between studies conducted in Asia/Africa and those conducted in North America/Europe. As discussed in paragraphs 4–6 of the Discussion, these differences may reflect variations in ADHD severity, environmental factors, or measurement methods.

To further examine these hypotheses, I suggest conducting the following additional analyses:

1. A meta-regression using the pooled ferritin level across the case and control groups as a moderator.

2. A meta-regression using ADHD severity as a moderator.

3. A subgroup analysis according to the diagnostic methods or assessment tools used to identify ADHD.

These analyses could help determine whether the observed heterogeneity is attributable to baseline ferritin status, ADHD severity, or differences in diagnostic and measurement approaches, thereby providing empirical support for the explanations proposed in the Discussion.

Reviewer #2: This systematic review and meta-analysis investigated the association between attention-deficit/hyperactivity disorder (ADHD) and serum ferritin levels. The study was conducted in accordance with the Preferred Reporting Items for Systematic Reviews and Meta-Analyses (PRISMA) guidelines. A total of 7,643 articles were initially identified, and 21 studies, comprising 4,058 children with ADHD and 4,533 healthy controls, were ultimately included in the meta-analysis. The pooled results indicated that lower serum ferritin levels were significantly associated with ADHD in children (pooled SMD: −9.74), suggesting that screening for serum ferritin levels and appropriate iron supplementation may be considered in this population.

Overall, this review was conducted rigorously, and the manuscript is well organized and clearly written. I have one minor comment. Several relevant studies appear not to have been included in the meta-analysis. The authors should clarify the reasons for their exclusion and explain whether these studies met the predefined eligibility criteria.

**********

what does this mean?). If published, this will include your full peer review and any attached files.

If you choose “no”, your identity will remain anonymous but your review may still be made public.

**Do you want your identity to be public for this peer review?**  For information about this choice, including consent withdrawal, please see our Privacy Policy

Reviewer #1: No

Reviewer #2: No

**********

[NOTE: If reviewer comments were submitted as an attachment file, they will be attached to this email and accessible via the submission site. Please log into your account, locate the manuscript record, and check for the action link "View Attachments". If this link does not appear, there are no attachment files.]

To ensure your figures meet our technical requirements, please review our figure guidelines: https://journals.plos.org/plosone/s/figures

You may also use PLOS’s free figure tool, NAAS, to help you prepare publication quality figures: https://journals.plos.org/plosone/s/figures#loc-tools-for-figure-preparation.

NAAS will assess whether your figures meet our technical requirements by comparing each figure against our figure specifications.

# Author comment: Author response to Decision Letter 1

**DOI:** 10.1371/journal.pone.0359790.r002

13 Sep 2026

Our responses to the editor and reviewer comments are provided in a separate file, labeled "Authors' Point-by-Point Response to Reviewers."

Attachment Submitted filename: Authors point by point response to reviewers.pdf

# Peer review: Decision Letter 1

**DOI:** 10.1371/journal.pone.0359790.r003

**Authors:** Mu-Hong Chen

17 Sep 2026

Serum Ferritin in Children with Attention Deficit Hyperactivity Disorder (ADHD): A Systematic Review and Meta-Analysis

PONE-D-26-33165R1

Dear Dr. Solomon Gedfie,

We’re pleased to inform you that your manuscript has been judged scientifically suitable for publication and will be formally accepted for publication once it meets all outstanding technical requirements.

Within one week, you’ll receive an e-mail detailing the required amendments. When these have been addressed, you’ll receive a formal acceptance letter and your manuscript will be scheduled for publication.

An invoice will be generated when your article is formally accepted. Please note, if your institution has a publishing partnership with PLOS and your article meets the relevant criteria, all or part of your publication costs will be covered. Please make sure your user information is up-to-date by logging into Editorial Manager at Editorial Manager® and clicking the ‘Update My Information' link at the top of the page. For questions related to billing, please contact billing support.

If your institution or institutions have a press office, please notify them about your upcoming paper to help maximize its impact. If they’ll be preparing press materials, please inform our press team as soon as possible -- no later than 48 hours after receiving the formal acceptance. Your manuscript will remain under strict press embargo until 2 pm Eastern Time on the date of publication. For more information, please contact onepress@plos.org.

Kind regards,

Mu-Hong Chen, M.D., Ph.D.

Academic Editor

PLOS One

Additional Editor Comments (optional):

Reviewers' comments:

Reviewer's Responses to Questions

**Comments to the Author**

Reviewer #1: All comments have been addressed

Reviewer #2: All comments have been addressed

**********

2. Is the manuscript technically sound, and do the data support the conclusions?

Reviewer #1: Yes

Reviewer #2: Yes

**********

3. Has the statistical analysis been performed appropriately and rigorously?

Reviewer #1: Yes

Reviewer #2: Yes

**********

4. Have the authors made all data underlying the findings in their manuscript fully available?

The PLOS Data policy

Reviewer #1: Yes

Reviewer #2: Yes

**********

5. Is the manuscript presented in an intelligible fashion and written in standard English?

Reviewer #1: Yes

Reviewer #2: Yes

**********

Reviewer #1: All comments have been addressed. I have no further comment for this paper

Reviewer #2: The article entitled "Serum Ferritin in Children with Attention Deficit Hyperactivity Disorder (ADHD): A

Systematic Review and Meta-Analysis" have substantially revised. The authors have adequately responded to my comments.

**********

what does this mean?). If published, this will include your full peer review and any attached files.

If you choose “no”, your identity will remain anonymous but your review may still be made public.

**Do you want your identity to be public for this peer review?**  For information about this choice, including consent withdrawal, please see our Privacy Policy

Reviewer #1: No

Reviewer #2: No

**********

# Editor report: Acceptance letter

**DOI:** 10.1371/journal.pone.0359790.r004

**Authors:** Mu-Hong Chen

PONE-D-26-33165R1

PLOS One

Dear Dr. Gedfie,

I'm pleased to inform you that your manuscript has been deemed suitable for publication in PLOS One. Congratulations! Your manuscript is now being handed over to our production team.

At this stage, our production department will prepare your paper for publication. This includes ensuring the following:

* All references, tables, and figures are properly cited

* All relevant supporting information is included in the manuscript submission,

* There are no issues that prevent the paper from being properly typeset

You will receive further instructions from the production team, including instructions on how to review your proof when it is ready. Please keep in mind that we are working through a large volume of accepted articles, so please give us a few days to review your paper and let you know the next and final steps.

Lastly, if your institution or institutions have a press office, please let them know about your upcoming paper now to help maximize its impact. If they'll be preparing press materials, please inform our press team within the next 48 hours. Your manuscript will remain under strict press embargo until 2 pm Eastern Time on the date of publication. For more information, please contact onepress@plos.org.

You will receive an invoice from PLOS for your publication fee after your manuscript has reached the completed accept phase. If you receive an email requesting payment before acceptance or for any other service, this may be a phishing scheme. Learn how to identify phishing emails and protect your accounts at https://explore.plos.org/phishing.

If we can help with anything else, please email us at customercare@plos.org.

Thank you for submitting your work to PLOS One and supporting open access.

Kind regards,

PLOS One Editorial Office Staff

on behalf of

Dr. Mu-Hong Chen

Academic Editor

PLOS One
